# Using the Five Domains Model to Assess the Adverse Impacts of Husbandry, Veterinary, and Equitation Interventions on Horse Welfare

**DOI:** 10.3390/ani8030041

**Published:** 2018-03-18

**Authors:** Paul McGreevy, Jeannine Berger, Nic de Brauwere, Orla Doherty, Anna Harrison, Julie Fiedler, Claudia Jones, Sue McDonnell, Andrew McLean, Lindsay Nakonechny, Christine Nicol, Liane Preshaw, Peter Thomson, Vicky Tzioumis, John Webster, Sarah Wolfensohn, James Yeates, Bidda Jones

**Affiliations:** 1 Sydney School of Veterinary Science, University of Sydney, New South Wales 2006, Australia; vicky.tzioumis@sydney.edu.au (V.T.); bjones@rspca.org.au (B.J.); 2 San Francisco SPCA, 201 Alabama Street, San Francisco, CA 94103, USA; jberger@sfspca.org; 3 Redwings Horse Sanctuary, Hapton, Norwich NR15 1SP, UK; nde-brauwere@redwings.co.uk; 4 Life Sciences Department, University of Limerick, Limerick V94 T9PX, Ireland; animalbehaviourclinic@gmail.com; 5 The Donkey Sanctuary, Sidmouth, Devon EX10 0NU, UK; anna.harrison@thedonkeysanctuary.org.uk; 6 Horse SA: 105 King William St, Kent Town 5067, Australia; horsesa@horsesa.asn.au; 7 RSPCA NSW, PO Box 34, Yagoona 2199, Australia; cjones@rspcansw.org.au; 8 University of Pennsylvania School of Veterinary Medicine, New Bolton Center, 382 W Street Rd, Kennett Square, PA 19348, USA; suemcd@vet.upenn.edu; 9 Equitation Science International, 3 Wonderland Ave, Tuerong 3915, Victoria, Australia; andrewmclean@esi-education.com; 10 Department of Animal Biosciences, University of Guelph, 50 Stone Road East, ON N1G 2W1, Canada; lnakonec@uoguelph.ca; 11 Royal Veterinary College, 4 Royal College St, Kings Cross, London NW1 OTU, UK; cnicol@rvc.ac.uk; 12 The Horse Trust, Slad Lane, Princes Risborough, Buckinghamshire HP27 OPP, UK; liane@horsetrust.org.uk; 13 School of Life and Environmental Sciences, University of Sydney, New South Wales 2006, Australia; peter.thomson@sydney.edu.au; 14 Bristol Veterinary School, University of Bristol, Langford BS40 5DU, UK; john.webster@bristol.ac.uk; 15 School of Veterinary Medicine, University of Surrey, Guildford, Surrey GU2 7XH, UK; s.wolfensohn@surrey.ac.uk; 16 RSPCA, Wilberforce Way, Southwater, Horsham, Sussex, RH13 9RS, UK; james.yeates@rspca.org.uk; 17 RSPCA Australia, P.O. Box 265, Deakin West, Australian Capital Territory 2600, Australia

**Keywords:** horse, welfare assessment, equitation, husbandry, five domains

## Abstract

**Simple Summary:**

Using an adaptation of the domain-based welfare assessment model, a panel of horse welfare professionals (with professional expertise in psychology, equitation science, veterinary science, education, welfare, equestrian coaching, advocacy, and community engagement) assessed the perceived harms, if any, resulting from 116 interventions that are commonly applied to horses. Scores for Domain 5 (the integrated mental impact) gathered after extensive discussion during a four-day workshop aligned well with overall impact scores assigned by the same panellists individually before the workshop, although some rankings changed after workshop participation. Domain 4 (Behaviour) had the strongest association with Domain 5, whilst Domain 1 (Nutrition) had the weakest association with Domain 5, implying that the panellists considered commonly applied nutritional interventions to have less of a bearing on subjective mental state than commonly applied behavioural restrictions. The workshop defined each intervention, and stated assumptions around each, resulting in a set of exemplar procedures that could be used in future equine welfare assessments.

**Abstract:**

The aim of this study was to conduct a series of paper-based exercises in order to assess the negative (adverse) welfare impacts, if any, of common interventions on domestic horses across a broad range of different contexts of equine care and training. An international panel (with professional expertise in psychology, equitation science, veterinary science, education, welfare, equestrian coaching, advocacy, and community engagement; *n* = 16) met over a four-day period to define and assess these interventions, using an adaptation of the domain-based assessment model. The interventions were considered within 14 contexts: C1 Weaning; C2 Diet; C3 Housing; C4 Foundation training; C5 Ill-health and veterinary interventions (chiefly medical); C6 Ill-health and veterinary interventions (chiefly surgical); C7 Elective procedures; C8 Care procedures; C9 Restraint for management procedures; C10 Road transport; C11 Activity—competition; C12 Activity—work; C13 Activity—breeding females; and C14 Activity—breeding males. Scores on a 1–10 scale for Domain 5 (the mental domain) gathered during the workshop were compared with overall impact scores on a 1–10 scale assigned by the same panellists individually before the workshop. The most severe (median and interquartile range, IQR) impacts within each context were identified during the workshop as: C1 abrupt, individual weaning (10 IQR 1); C2 feeding 100% low-energy concentrate (8 IQR 2.5); C3 indoor tie stalls with no social contact (9 IQR 1.5); C4 both (i) dropping horse with ropes (9 IQR 0.5) and forced flexion (9 IQR 0.5); C5 long-term curative medical treatments (8 IQR 3); C6 major deep intracavity surgery (8.5 IQR 1); C7 castration without veterinary supervision (10 IQR 1); C8 both (i) tongue ties (8 IQR 2.5) and (ii) restrictive nosebands (8 IQR 2.5); C9 ear twitch (8 IQR 1); C10 both (i) individual transport (7.00 IQR 1.5) and group transport with unfamiliar companions (7 IQR 1.5); C11 both (i) jumps racing (8 IQR 2.5) and Western performance (8 IQR 1.5); C12 carriage and haulage work (6 IQR 1.5); C13 wet nurse during transition between foals (7.5 IQR 3.75); and C14 teaser horse (7 IQR 8). Associations between pre-workshop and workshop scores were high, but some rankings changed after workshop participation, particularly relating to breeding practices. Domain 1 had the weakest association with Domain 5. The current article discusses the use of the domain-based model in equine welfare assessment, and offers a series of assumptions within each context that future users of the same approach may make when assessing animal welfare under the categories reported here. It also discusses some limitations in the framework that was used to apply the model.

## 1. Introduction

The concept of ethical use of animals is illustrated by the United Kingdom (UK) Scientific Procedures Act [1], which introduced a systematic approach to the evaluation of harms and benefits in relation to the use of experimental animals (harm:benefit analysis, HBA). Under this legislation, each application to carry out a regulated procedure on a protected animal must include: an estimation of the likely severity of the procedure, which is defined as mild, moderate, or substantial; the steps taken to minimise severity (e.g., use of anaesthetics); and the likely benefit to humans, other animals, or the environment. HBA is most often conducted prospectively by trying to estimate future benefits and harms, but once procedures or interventions have been conducted, it is also possible to use retrospective data to assess the actual harms and benefits [2].

While we do not propose that all of the elective procedures or other interventions with horses should be subject to a similar degree of bureaucratic control, the principles that underpin HBA are highly applicable to the interventions that we elect to impose on horses in our care.

It is self-evident that all elective processes and interventions are undertaken for a reason. However, if we are to justify a procedure, we must establish that the reason is both good and sufficient. In some cases, we may question whether the procedure is even necessary. The aim of the current project was to assess the potential harmful impacts of common interventions on horses. This information could be used in the future alongside knowledge of the nature and extent of possible benefits accruing from each intervention in order to justify (or otherwise) these procedures for any species within our care.

Animal welfare science has identified a large number of objective measures that can be used to determine the welfare state of an animal; there is no single measure or standard that can be used by itself to tell us an animal’s state [3,4,5]. Some approaches assess how animals cope with the challenges they face as reflected in the normality of their biological functioning and fitness [6]. Other approaches rely on behavioural observations (see Dawkins 2004) to capture both the physical and mental aspects of an animal’s welfare state. A key challenge is to ascertain what matters from the animal’s point of view [7]. This can be done by recording animals’ choices and decisions and using these to provide an insight into affective states [8]. Other welfare measures can be mapped onto these expressed preferences to give greater confidence in the welfare assessment processes (e.g., [9]). However, such experimental approaches are expensive and time-consuming.

When multiple measures are available (and when these do not always provide a consistent picture), expert opinion can be used to decide which are the most important or informative indicators, and draw integrated conclusions [10,11,12,13,14]. When considering not only the short-term welfare state, but also the animal’s quality of life over a longer period [15,16,17] or whether overall it has a “life worth living” [18], expert opinion is then often the best or only tool available to develop a picture of the overall welfare impact of a given procedure of intervention in an animal’s life.

The aim of this project was to conduct a series of assessments of the negative (adverse) welfare impacts of the common interventions that are applied to domestic horses across a broad range of different contexts of equine care and training. For the first time, an international panel assessed the harmful impacts, if any, of interventions using an adaptation [19] of the domain-based model [20]. We also explored the consistency with which Domain 5, the mental domain, was assessed by a panel before and after extensive workshop discussion. The current article may facilitate the development of a scoring system that allows horse carers to map the welfare impacts on horses of various management, training, and competition activities.

The panel completed a pre-workshop survey, and then spent four days assessing the adverse welfare impact on horses [horses and ponies] of common interventions across a broad range of contexts [things we do to domesticated horses] using the Five Domains model of animal welfare assessment [21] as a framework. The Five Domains have been used as the basis of a severity scale to systematically assess and record the level of welfare compromise to animals used in research, teaching, and testing procedures in New Zealand since 1997 [22,23]. Expert panels have also previously used the Five Domains model to assess the impact of vertebrate pest control methods [24,25]. There is potential for this approach to be extended to other domesticated and captive species.

## 2. Method

### 2.1. Panellist Recruitment and Selection

For the current workshop, 16 invited panellists from Australia, the United Kingdom, Ireland, Canada, and the United States were recruited to represent a diverse range of equine-related professionals with expertise in psychology, equitation science, veterinary science, education, welfare, equestrian coaching, advocacy, and community engagement. All of the delegates, who were recruited back on a track record of having worked on horse-welfare issues, accepted an emailed invitation to join the panel. A mix of veterinarians and laypersons ensured that differing points of view were presented.

### 2.2. Pre-Workshop Survey

Pre-workshop planning enabled a maximum value of available face-to-face time for the assessment of each intervention. The panellists were asked individually to propose various husbandry, equitation, and veterinary interventions applied to horses. From these suggestions, a comprehensive list of interventions (*n* = 196) was assembled. We considered these 196 interventions within 14 broad contexts, namely: C1 Weaning; C2 Diet; C3 Housing; C4 Foundation training; C5 Ill-health and veterinary interventions (chiefly medical); C6 Ill-health and veterinary interventions (chiefly surgical); C7 Elective procedures; C8 Care procedures; C9 Restraint for management procedures; C10 Road transport; C11 Activity—competition; C12 Activity—work; C13 Activity—breeding females; and C14 Activity—breeding males. Excluding the two contexts of ill health, horses were assumed to be healthy in all other contexts.

Following the suggestion phase, panellists were sent the full list of 196 interventions for a pre-workshop survey. Panellists were asked to express their self-nominated levels of interest and knowledge in each context. This enabled us to identify the preferred workshop leaders for each context, and establish which contexts would be considered by all panellists, and which would be considered by a subset. The pre-workshop survey also asked each panellist to provide a single adverse impact score for each procedure. The respondents used a 10-point scale to score the overall impact of each intervention on horse welfare, from “No impact” (1) to “Extreme impact” (10) (plus “Don’t Know”).

Before the workshop, each context leader supplied an overview of the context, as well as annotated references, to support welfare assessments during the workshop. This was distributed to the panellists as a handbook.

### 2.3. Workshop

The list of interventions (*n* = 196) was considered over the course of the four-day workshop, which allowed each context to be discussed in a discrete session. Of these, 60 were eliminated because the intervention was unfamiliar to five or more panellists, or because panellists agreed that they were either: too broad (e.g., fencing); outdated or illegal (e.g., docking); covered by other interventions (e.g., it was decided that the training required for competitive equestrian activities should be considered as an integrated part of those activities, rather than separately); likely to be of negligible impact (e.g., hogging manes), or for which time was unavailable (e.g., all permutations of indoor stabling, levels of social contact, and degrees and conditions of turn-out to paddock). To explain the absence of interventions that some readers may regard as common or salient, the eliminated interventions and the reasons for their elimination are presented in Appendix A Table A1. A further 27 interventions were either modified in order to ensure a specific focus (Appendix A Table A2), combined (e.g., junior (Pony Club) and adult riding competitive activities were combined under community club competition), or split (e.g., diet at pasture was considered where there was choice and no choice). A total of 116 interventions remained after the process of elimination and modification; these interventions were subsequently discussed during the workshop.

While donkeys and the significant differences (between horses and ponies) regarding their anatomy, physiology, and behavioural needs were discussed regularly at the workshop, the scoring exercise was ultimately conducted for horses, and the results presented here do not apply directly to donkeys.

At the start of the workshop, panellists were provided with:

- Assessment spreadsheets (those with laptops/tablets used these to enter their assessment scores; those without such technology used paper forms). The format of the assessments sheets’ spreadsheets was the same as the tables that appear in Supplementary Materials;
- The International Society for Equitation Science (ISES) position statement on aversive stimuli [26];
- The Five Domains poster [21]; and
- *Horse Sense* [27] and *Equine Clinical Medicine: Surgery and Reproduction* [28] (one copy per two panellists).

These materials were selected primarily because the organizers (PM and BJ) were familiar with them, having used them in previous teaching and policy development work. The workshop proceeded with sessions that considered each context.

For five sessions, the panel sat as a whole, and for the remaining five sessions, it split into two subpanels. Panellists had nominated their particular expertise and interest ahead of the workshop, and were assigned to subpanels accordingly in advance. The pairs of subpanels were: road transport and foundation training; medical interventions and activity (work); activity (competition—part 1) and surgical interventions; weaning and activity (breeding stallions); activity (breeding mares); and activity (competition—part 2). At the start of each session, the context leader introduced the potential welfare implications pertaining to each context, and identified gaps in the literature. Next, the interventions were more closely defined, and assumptions around each were delineated.

### 2.4. Assumptions

The panellists were required to agree on a series of assumptions before estimating the impact of each intervention. For every cluster of interventions, we asked panellists to assume certain conditions before the workshop. Where no such details were supplied, panellists were asked to assume best practice or standard practice. At the workshop, the pre-workshop assumptions were discussed in some detail and expanded and refined. The term “best practice” was discussed, and textbooks were provided to identify standard practice in the hands of a competent practitioner. We agreed to consider best practice as it is currently done, rather than the best possible practice.

The details of these assumptions (including pre-workshop assumptions, if they were provided to the panellists for the pre-workshop survey) appear in the shaded sections of Tables S1–S14. They are presented here for two reasons. First, they may help to explain the reasons behind the impact scores that appear in the Results section, and second, they may assist future efforts in similar activities.

### 2.5. Assessment of Adverse Welfare Impact

**Figure 1:**
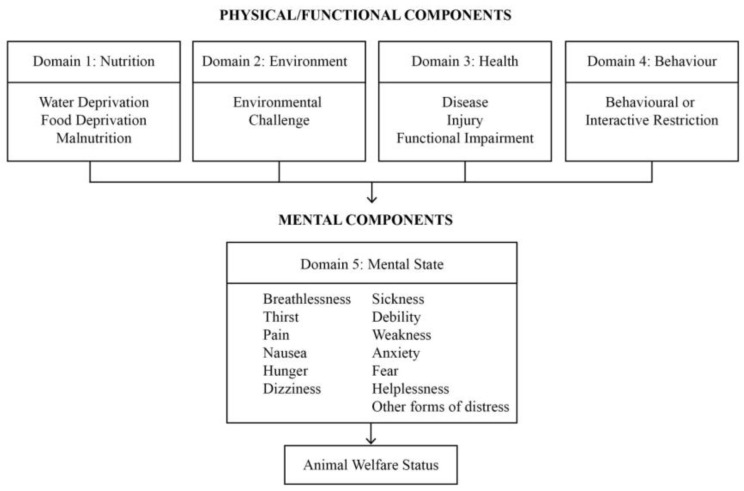
Domains of potential welfare compromise divided broadly into physical or functional and mental components. (Modified from Mellor et al., 2009 [24]).

The assessment approach for this workshop was based on the Five Domains model developed by Mellor and Reid [20] and updated over time, based on emerging behavioural, physiological, and neuroscientific evidence [19,23] (see Figure 1).

This model has been adapted to provide a practical means of assessing negative or adverse welfare impacts on animals in several different areas, most notably to assess the impact of vertebrate pest control methods [24,29]. Jones and McGreevy first proposed the method as a means of evaluating the welfare impact of interventions on horses [30], and operational details of the model’s use have been published recently [31].

The approach taken by Sharp and Saunders involves the use of an expert panel to reach consensus on the welfare compromise of different methods of vertebrate pest control. The panel approach provides a way for individuals with different experiences regarding each intervention (animal welfare scientists, practitioners, veterinarians, etc.) to define the intervention, and discuss each domain before reaching consensus on the overall welfare impact. We used this process at the workshop to assess multiple interventions to horses across the 14 different contexts. However, we did not seek consensus. Instead, the panellists scored the impact of each intervention privately.

Under the Five Domains model, potential or actual welfare compromise is identified in four physical or functional domains and one mental domain, which are numbered 1 to 5. When considering a procedure or intervention, panellists used their knowledge and relevant literature to appraise the potential compromise relating to each domain.

In the context of welfare compromise, the first four domains focus on internal physiological and pathophysiological disturbances due to nutritional, environmental, and health-related problems (Domains 1–3), and external physical, biotic, and social conditions in the animal’s environment that may limit its capacity to express various behaviours or may otherwise pose significant challenges (Domain 4) [23]. At the workshop, once the discussion regarding each intervention had ended, panellists privately scored the impact under each of the five domains, with Domain 5 being an assessment of the overall impact on the mental state of the animals in question.

The scores of 15 panellists are reported here. The 16th panellist acted as a scribe and workshop facilitator, referring to textbooks during each session. Some panellists elected to abstain from scoring specific contexts if they had been absent from a critical element of the workshop discussion. The number of panellists scoring each context is given in the Results section.

We analysed the differences between the pre-workshop overall impact scores for each intervention, and the workshop scores for Domain 5.

### 2.6. Statistical Analysis

Those who analyzed the scores of the panellists were blinded to panellists’ identity. As the data were scored on an ordinal scale, descriptive statistics were provided as boxplots to show medians, quartiles, and outliers.

We were interested in the factors that predicted the overall Domain 5 score that the panellists allocated during the workshop. We focussed on Domain 5 because it arguably had the most direct relevance to the animals’ welfare state. We then conducted an analysis to determine whether the pre-workshop scores were associated with the ultimate Domain 5 scores that were allocated during the workshop. It is important to note that the panellists scored interventions relative to each other within each context, and no attempt was made to standardise scoring across the 14 different contexts. Thus, a score of seven in the care context, for example, may not have been equivalent to a score of seven in the surgical context. For this reason, we produced a series of 14 separate logistic regression models [32] in R studio [33]: one for each context.

We also examined which (if any) of the scores allocated to the physical Domains 1, 2, 3, and 4 were most strongly associated with the overall Domain 5 score. This was repeated (as above) for each of the 14 contexts, potentially producing a maximum of 56 analyses. However, for some context × domain combinations, all of the domain scores were equal; hence, no association could be tested in these situations.

As the data were scored on an ordinal scale, an ordinal logistic generalised linear mixed model (GLMM) was used. The following ordinal mixed model was fitted to the data for each possible context × domain combination:

$$\log_{e}\left(\frac{P(Y\leq k)}{P(Y>k)}\right)=\theta_{k}-({\mathrm{Score}}_{i}+\mathrm{Participant})$$

where

*Y* = Domain 5 score (0, 1, …, 9);
θ*_k_* = ordered intercept for Domain 5 score k;
Score*_i_* = effect Domain Score i (*i* = 1, …, 4), or pre-workshop score; and
Participant = random effect of the participant.

The models were fitted using the ‘clmm’ function of the ordinal package [33]. Significance testing was made using likelihood ratio tests. Model-based probabilities were obtained from the fitted model, and used to illustrate the findings.

## 3. Results

### 3.1. Descriptive Statistics

Boxplots providing the panellists’ scores for each context are shown in Figure 2, Figure 3, Figure 4, Figure 5, Figure 6, Figure 7, Figure 8, Figure 9, Figure 10, Figure 11, Figure 12, Figure 13, Figure 14 and Figure 15.

**Figure 2:**
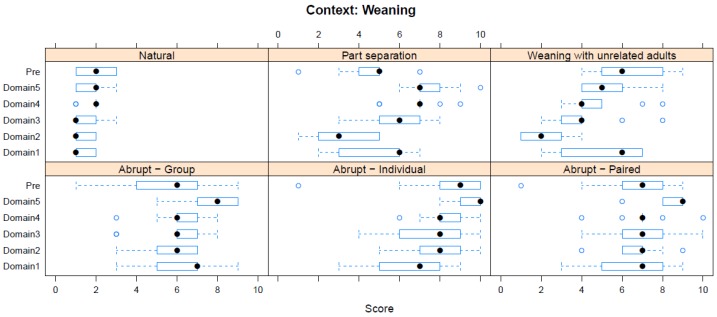
Box-and-whisker plots showing the distribution of pre-workshop (Pre) estimates (where available) of the impact of various weaning interventions, and the workshop scores assigned to these interventions using the Five Domains approach. The pre-workshop estimates were not based on the Five Domains approach. For the assumptions and notes on which these domain scores were based, see Supplementary Materials Table S1.

**C1 Weaning** (Figure 2) (panellists *n* = 9)

For Weaning, abrupt individual weaning was scored as having the greatest adverse impact in both pre-workshop (median score 9, IQR 2) and workshop (Domain 5 median score 10, IQR 1) assessments. The lowest scores were for natural weaning (pre-workshop median score 2, IQR 1; workshop, Domain 5 median score 2, IQR 1).

**Figure 3:**
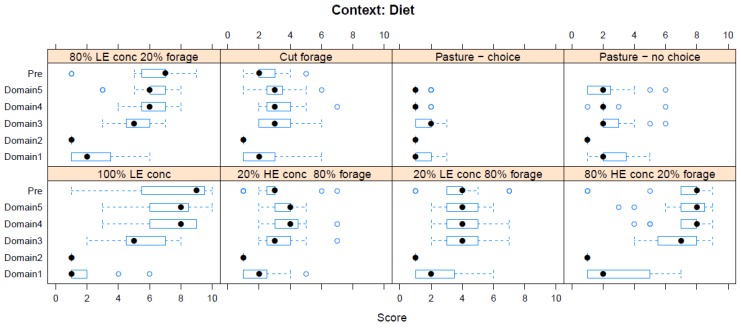
Box-and-whisker plots showing the distribution of pre-workshop (Pre) estimates (where available) of the impact of various dietary interventions, and the workshop scores assigned to these interventions using the Five Domains approach. The pre-workshop estimates were not based on the Five Domains approach. For the assumptions and notes on which these domain scores were based, see Supplementary Materials Table S2.

**C2 Diet** (Figure 3) (panellists *n* = 15)

For Diet, feeding 100% low-energy (LE) concentrate was scored as having the greatest adverse impact in both pre-workshop (median score 9, IQR 3) and workshop (Domain 5 median score 8, IQR 2.5) assessments. This was not because it was felt to have an adverse effect on nutrition (Domain 1 scores were low), but rather because of its adverse effect on behaviour (Domain 4). The lowest scores were for cut forage (pre-workshop median score 2, IQR 1) or varied pasture with choice of grazing and browsing (workshop, Domain 5 median score 2, IQR 1; this option was not available pre-workshop).

**Figure 4:**
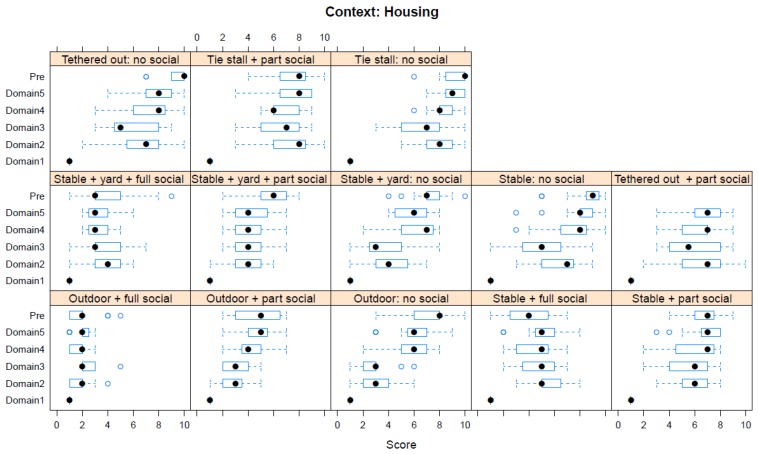
Box-and-whisker plots showing the distribution of pre-workshop (Pre) estimates (where available) of the impact of various housing interventions, and the workshop scores assigned to these interventions using the Five Domains approach. The pre-workshop estimates were not based on the Five Domains approach. For the assumptions and notes on which these domain scores were based, see Supplementary Materials Table S3.

**C3 Housing** (Figure 4) (panellists *n* = 15)

For Housing, the pre-workshop scores identified both (i) outdoor tethering with no social contact (median score 10, IQR 1) and indoor tie stalls with no social contact (median score 10, IQR 2) as having the greatest adverse impacts. During the workshop, indoor tie stalls with no social contact (Domain 5 median score 9, IQR 1.5) were assessed as having the greatest adverse impact. The lowest scores were for living outdoors with full social contact (pre-workshop median score 2, IQR 1; workshop, Domain 5 median score 2, IQR 0.5).

**Figure 5:**
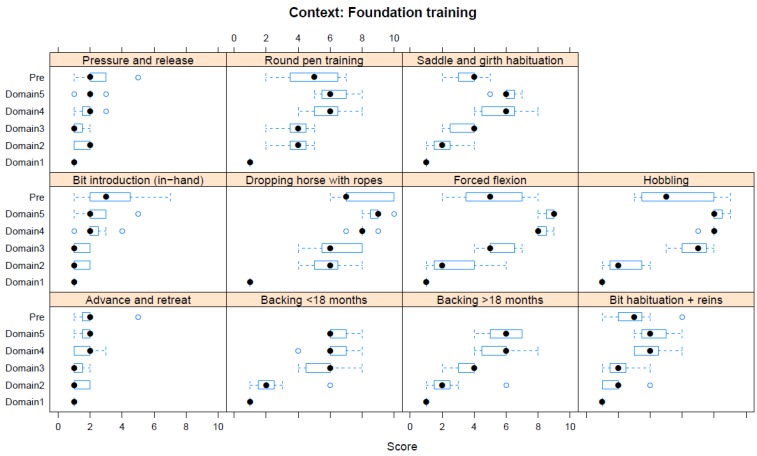
Box-and-whisker plots showing the distribution of pre-workshop (Pre) estimates (where available) of the impact of various foundation training interventions, and the workshop scores assigned to these interventions using the Five Domains approach. The pre-workshop estimates were not based on the Five Domains approach. For the assumptions and notes on which these domain scores were based, see Supplementary Materials Table S4.

**C4 Foundation Training** (Figure 5) (panellists *n* = 7)

For Foundation Training, the pre-workshop scores identified dropping a horse with ropes as having the greatest adverse impact (median score 7, IQR 3). During the workshop, both dropping a horse with ropes and forced flexion (each Domain 5 median score 9, IQR 0.5) were assessed as having the greatest adverse impact. The lowest scores were for both advance/retreat methods (pre-workshop median score 2, IQR 1; workshop, Domain 5 median score 2, IQR 0.5) and pressure/release methods (pre-workshop median score 2, IQR 1; workshop, Domain 5 median score 2, IQR 0). A greater divergence in scores for Domains 1 to 4 was noted for this context, with adverse scores primarily relating to behaviour and (in some cases, e.g., hobbling) to health.

**Figure 6:**
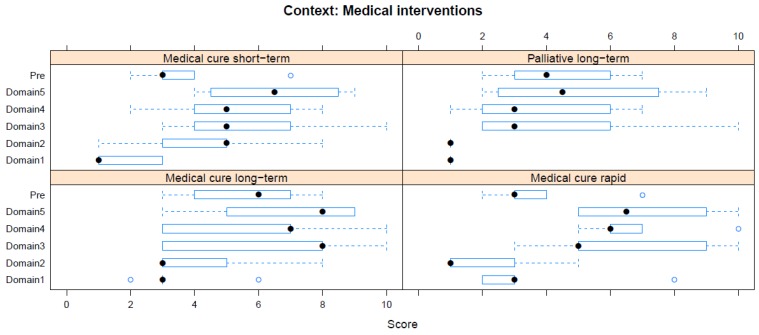
Box-and-whisker plots showing the distribution of pre-workshop (Pre) estimates (where available) of the impact of various medical interventions, and the workshop scores assigned to these interventions using the Five Domains approach. The pre-workshop estimates were not based on the Five Domains approach. For the assumptions and notes on which these domain scores were based, see Supplementary Materials Table S5.

**C5 Ill-Health and Veterinary Interventions (chiefly medical)** (Figure 6) (assessed by subpanel, panellists *n* = 4–5)

For Medical Interventions, the pre-workshop scores identified long-term curative medical treatments as having the greatest adverse impact (median score 6, IQR 3). This was also the case during the workshop, but with an increased average score (Domain 5 median score 8, IQR 3). The lowest pre-workshop scores were for immediately or short-term curative treatments (median score 3, IQR 1 in both cases). Scores for these increased markedly following workshop discussion (see Figure 6), such that the lowest ranked intervention assessed during the workshop was long-term palliative treatment (Domain 5 median score 4.5, IQR 4). Variation between panellists was higher for this context than most of the others.

**Figure 7:**
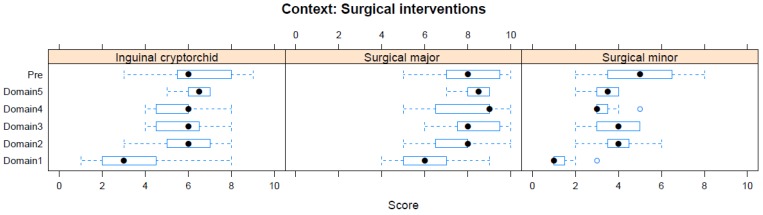
Box-and-whisker plots showing the distribution of pre-workshop (Pre) estimates (where available) of the impact of various surgical interventions, and the workshop scores assigned to these interventions using the Five Domains approach. The pre-workshop estimates were not based on the Five Domains approach. For the assumptions and notes on which these domain scores were based, see Supplementary Materials Table S6.

**C6 Ill-Health and Veterinary Interventions** (chiefly surgical) (Figure 7) (assessed by subpanel, panellists *n* = 6–7)

For Surgical Interventions, major deep intracavity surgery was scored as having the greatest adverse impact in both pre-workshop (median score 8, IQR 2.5) and workshop (Domain 5 median score 8.5, IQR 1) assessments. Single minor surgical intervention received the lowest score during pre-workshop (median score 5, IQR 3) and workshop (Domain 5 median score 3.5, IQR 1) assessments.

**Figure 8:**
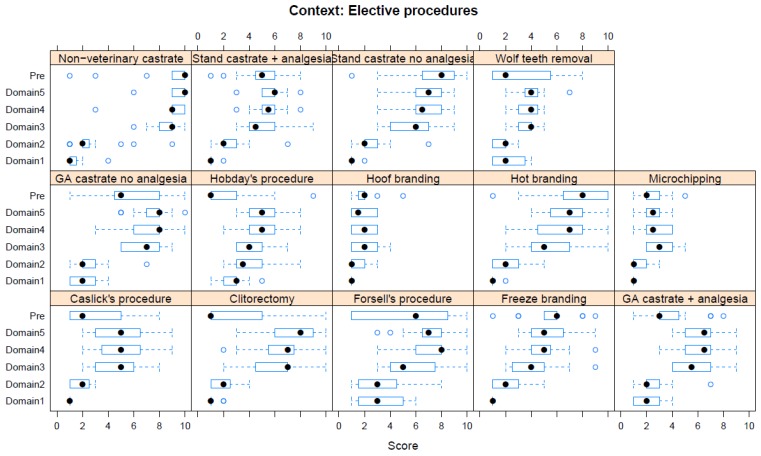
Box-and-whisker plots showing the distribution of pre-workshop (Pre) estimates (where available) of the impact of various elective procedures, and the workshop scores assigned to these procedures using the Five Domains approach. The pre-workshop estimates were not based on the Five Domains approach. For the assumptions and notes on which these domain scores were based, see Supplementary Materials Table S7.

**C7 Elective Procedures** (Figure 8) (panellists *n* = 14–15)

For Elective Procedures, castration without veterinary supervision was scored as having the greatest adverse impact in both pre-workshop (median score 10, IQR 1) and workshop (Domain 5 median score 10, IQR 1) assessments. The lowest scores during the pre-workshop assessments were given for clitorectomy (median score 1, IQR 4) and the Hobday procedure (median score 1, IQR 2). Scores for these procedures increased markedly following workshop discussion (see Fig 8). The lowest score given during the workshop assessment was for hoof branding (Domain 5 median score 1.5, IQR 2).

**Figure 9:**
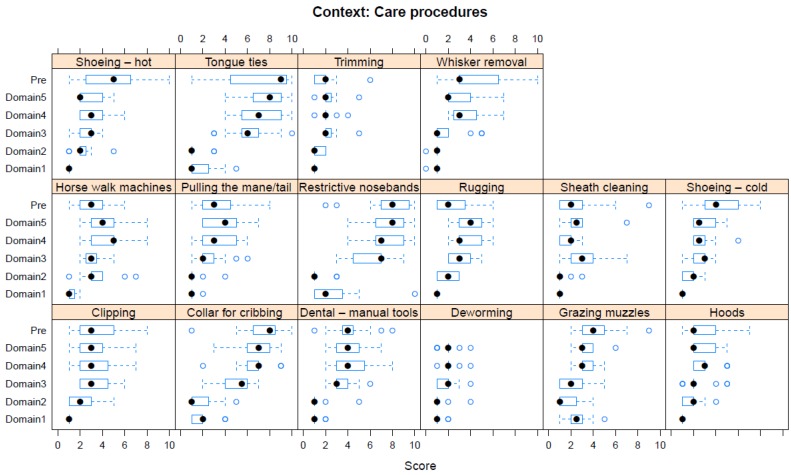
Box-and-whisker plots showing the distribution of pre-workshop (Pre) estimates (where available) of the impact of various care procedures, and the workshop scores assigned to these procedures using the Five Domains approach. The pre-workshop estimates were not based on the Five Domains approach. For the assumptions and notes on which these domain scores were based, see Supplementary Materials Table S8.

**C8 Care Procedures** (Figure 9) (panellists *n* = 14–15)

For Care Procedures, tongue ties were scored as having the greatest adverse impact in both pre-workshop (median score 9, IQR 5) and workshop (Domain 5 median score 8, IQR 2.5) assessments. Additionally, restrictive nosebands were scored as having an equally high adverse impact during the workshop assessment (Domain 5 median score 8, IQR 2.5). Low median scores of two were given pre-workshop for wearing hoods, rugging, sheath cleaning, and trimming; while during the workshop, low Domain 5 median scores of 2 were given for deworming, hot shoeing, trimming, and whisker removal.

**Figure 10:**
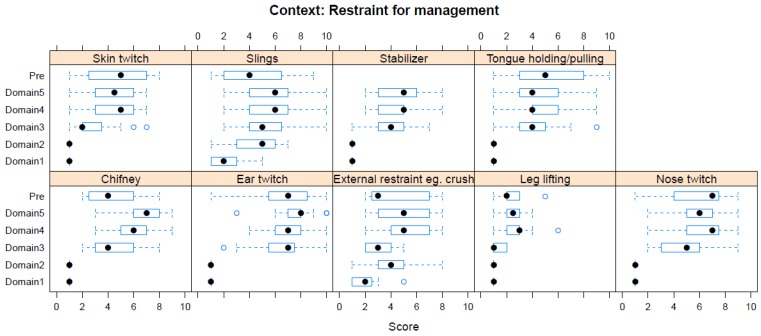
Box-and-whisker plots showing the distribution of pre-workshop (Pre) estimates (where available) of the impact of various restraint interventions, and the workshop scores assigned to these interventions using the Five Domains approach. The pre-workshop estimates were not based on the Five Domains approach. For the assumptions and notes on which these domain scores were based, see Supplementary Materials Table S9.

**C9 Restraint for Management Procedures** (Figure 10) (panellists *n* = 14–15)

For Restraint procedures, pre-workshop (median score 7, IQR 3) and workshop (Domain 5 median score 8, IQR 1) scores identified ear twitch as having the greatest adverse effect. The pre-workshop scoring also identified nose twitch in this regard (median score 7, IQR 3.5). The restraint intervention with the lowest pre-workshop (median score 2, IQR 1) and workshop (Domain 5 median score 2, IQR 1) score was leg-lifting.

**Figure 11:**
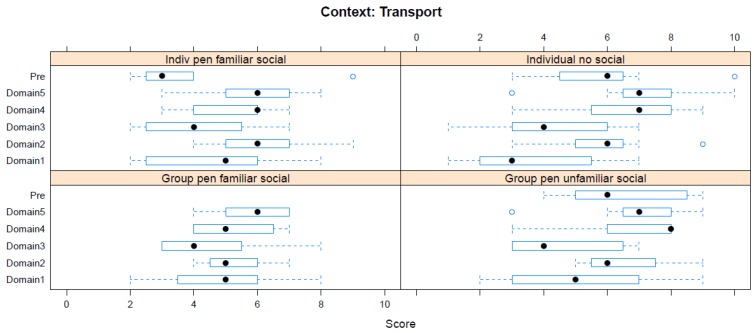
Box-and-whisker plots showing the distribution of pre-workshop (Pre) estimates (where available) of the impact of various road transport interventions, and the workshop scores assigned to these interventions using the Five Domains approach. The pre-workshop estimates were not based on the Five Domains approach. For the assumptions and notes on which these domain scores were based, see Supplementary Materials Table S10.

**C10 Road Transport** (Figure 11) (panellists *n* = 7)

For Road Transport procedures, the pre-workshop scores identified both transport in a group with unfamiliar companions (median score 6, IQR 3.5) and individual transport (median score 6, IQR 2) as interventions with the greatest impact in this context. The workshop scores similarly ranked these two interventions as having the greatest impact (both Domain 5 median scores 7, IQR 1.5). Travelling in an individual pen accompanied by familiar individuals was given the lowest pre-workshop score (median score 3, IQ 1.5). The workshop assessment agreed that this had the least harmful impact of the interventions considered, but the score increased (Domain 5 median score 6, IQR 2). The same score was given for travelling in a group pen with familiar individuals (this option not available pre-workshop). Road transport scored relatively highly for an adverse environment (Domain 2) in comparison with other contexts.

**Figure 12:**
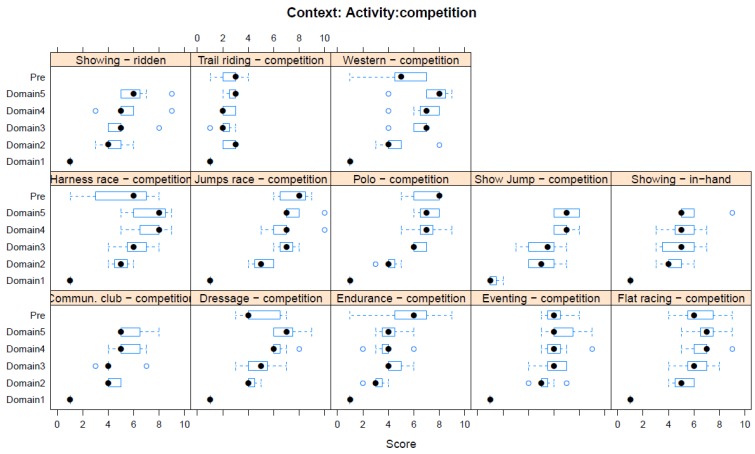
Box-and-whisker plots showing the distribution of pre-workshop (Pre) estimates (where available) of the impact of various competition interventions, and the workshop scores assigned to these interventions using the Five Domains approach. The pre-workshop estimates were not based on the Five Domains approach. For the assumptions and notes on which these domain scores were based, see Supplementary Materials Table S11.

**C11 Activity—Competition** (Figure 12) (panellists *n* = 7)

For Competitive Activities, the pre-workshop scores for adverse impact were highest for both jumps racing (8 IQR 2) and (ii) polo competition (8 IQR 2); whilst the highest workshop scores were for both jumps racing (8 IQR 2.5) and Western performance (8 IQR 1.5). The competitive activity that panellists assessed as having the lowest adverse impact was trail riding (pre-workshop median score 3, IQR 1; workshop, Domain 5 median score 3, IQR 0.5).

**Figure 13:**
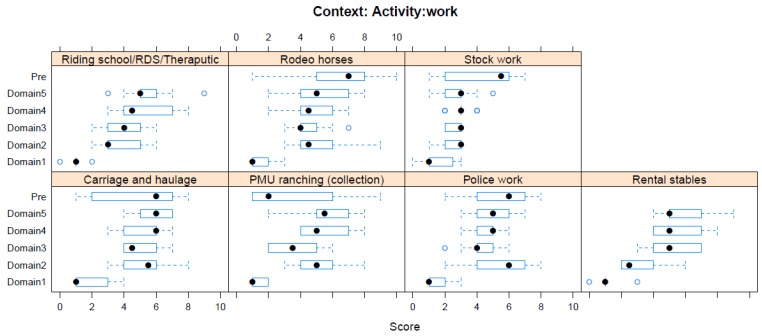
Box-and-whisker plots showing the distribution of pre-workshop (Pre) estimates (where available) of the impact of various work interventions, and the workshop scores assigned to these interventions using the Five Domains approach. The pre-workshop estimates were not based on the Five Domains approach. For the assumptions and notes on which these domain scores were based, see Supplementary Materials Table S12.

**C12 Activity—Work** (Figure 13) (panellists *n* = 10)

For Work activities, the pre-workshop scoring exercise identified rodeo as the activity with the greatest adverse impact (median score 7, IQR 2.5), whilst during the workshop, carriage and haulage work was considered to have the greatest adverse impact (Domain 5 median score 6, IQR 1.5). The pre-workshop assessment rated the collection of urine from pregnant mares (PMU) to have a low adverse impact (median score 2, IQR 4.5), but this score increased markedly after workshop discussion (see Figure 13). During the workshop, stock work was assessed as having the lowest score within this context (Domain 5 median score 3, IQR 0.75), and showed a marked decrease in score compared with pre-workshop assessments.

**Figure 14:**
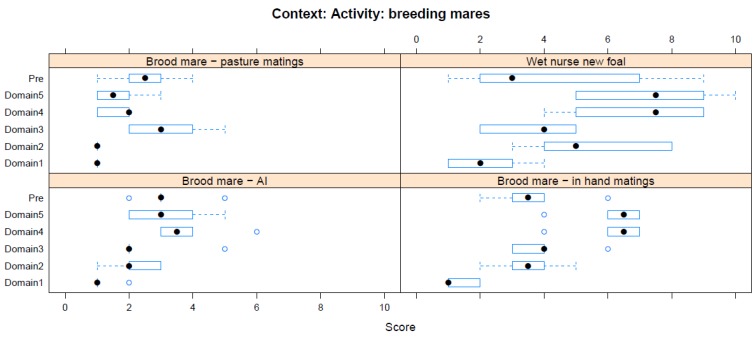
Box-and-whisker plots showing the distribution of pre-workshop (Pre) estimates (where available) of the impact of various interventions with breeding mares, and the workshop scores assigned to these interventions using the Five Domains approach. The pre-workshop estimates were not based on the Five Domains approach. For the assumptions and notes on which these domain scores were based, see Supplementary Materials Table S13.

**C13 Activity—Breeding Mares** (Figure 14) (panellists *n* = 6)

For breeding activities affecting mares, in-hand mating was identified as having the greatest impact in the pre-workshop scoring (median score 3.5, IQR 1), whilst during the workshop, wet nursing during transition between foals was considered to have a far greater adverse impact (Domain 5 median score 7.5, IQR 3.75). Pasture mating was considered to be the intervention with the least harmful impact (pre-workshop median score 2.5, IQR 1; workshop Domain 5 median score 1.5, IQR 1).

**Figure 15:**
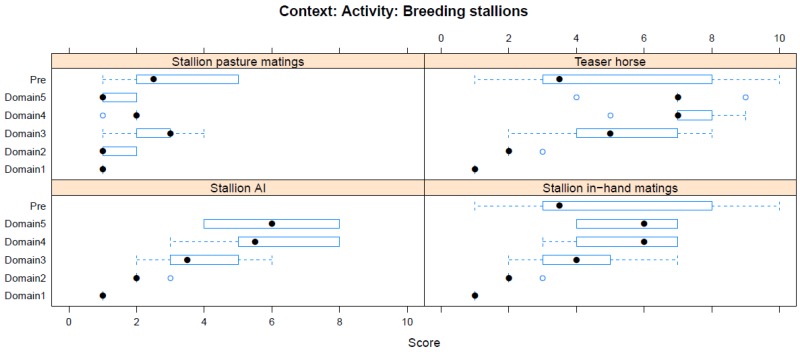
Box-and-whisker plots showing the distribution of pre-workshop (Pre) estimates (where available) of the impact of various interventions with breeding stallions (and teasers), and the workshop scores assigned to these interventions using the Five Domains approach. The pre-workshop estimates were not based on the Five Domains approach. For the assumptions and notes on which these domain scores were based, see Supplementary Materials Table S14.

**C14 Activity—Breeding Males** (Figure 15) (panellists *n* = 5–6)

For breeding activities affecting males, the experiences of teaser males and stallion in-hand matings were both rated most highly within this context (both median scores 3.5, IQR 4). During the workshop, the experiences of teaser males were ranked as the greatest adverse impact within this category, with scores that were higher (but highly variable between panellists) than during the pre-workshop assessment (Domain 5 median score 7, IQR 8). Pasture mating was considered to be the intervention with least harmful impact (pre-workshop median score 2.5, IQR 2.5; workshop Domain 5 median score 2.5, IQR 2.5).

### 3.2. Associations with Domain 5

#### 3.2.1. Pre-workshop vs. Domain 5

Associations between the pre-workshop scores and Domain 5 scores are shown in the final column of Table 1.

Significant associations were evident in most contexts, but there were Significant associations were evident in most contexts, but there were no significant associations between pre-workshop scores and Domain 5 scores for the medical and surgical interventions (scored by a smaller number of panellists), or the activity of breeding mares. There were general increases in Domain 5 scores with increasing pre-workshop scores, as shown in the table of regression coefficients (Table 2). For some contexts, e.g., housing, a steady increase was seen. For other contexts, e.g., work activities, the general pattern was more variable.

**Figure 16:**
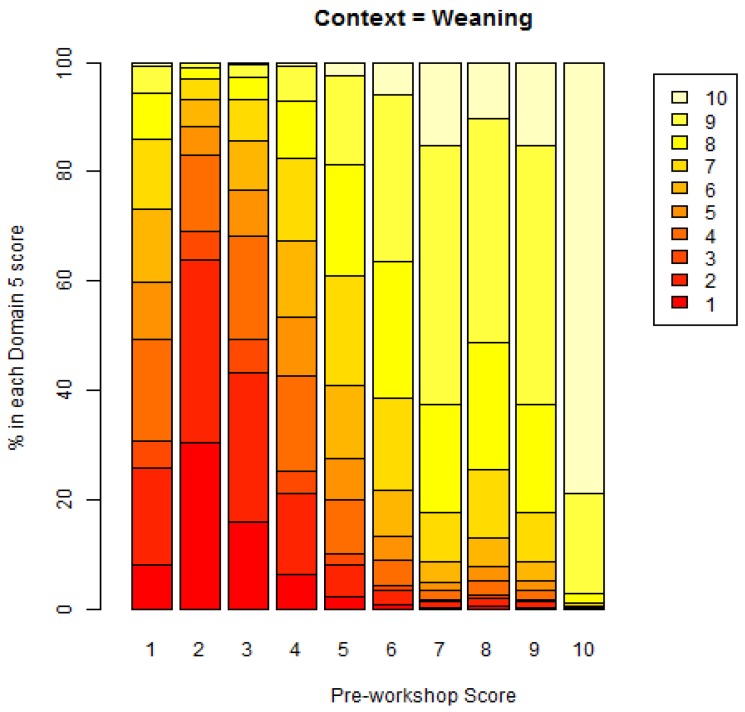
Model-based percentages of each Domain 5 score versus the pre-workshop score, for Weaning context.

A graphical example showing the pattern of association is given for the context of weaning in Figure 16 below. The predictions of the model based on the ordinal GLMM regression output are shown visually. For example, on 80% of occasions, a pre-workshop score of 10 would also result in a Domain 5 score of 10, with a very low probability of a Domain 5 score of less than 9.

#### 3.2.2. Domains 1 to 4 vs. Domain 5

The strength of associations between Domain 5 scores and those for Domains 1–4 are shown in Table 1. In most context × domain combinations, the associations were highly significant. This was particularly the case for Domain 3 (Health) and Domain 4 (Behaviour) in all contexts. Positive associations between Domain 1 (Nutrition) and Domain 5 occurred only in some contexts, e.g., weaning. Where significant associations existed, regression coefficient estimates showed that increasing scores for a particular domain were associated with an increased probability of a higher Domain 5 score. Examples of these patterns of positive associations for weaning, diet, and housing appear in Table 3).

**Figure 17:**
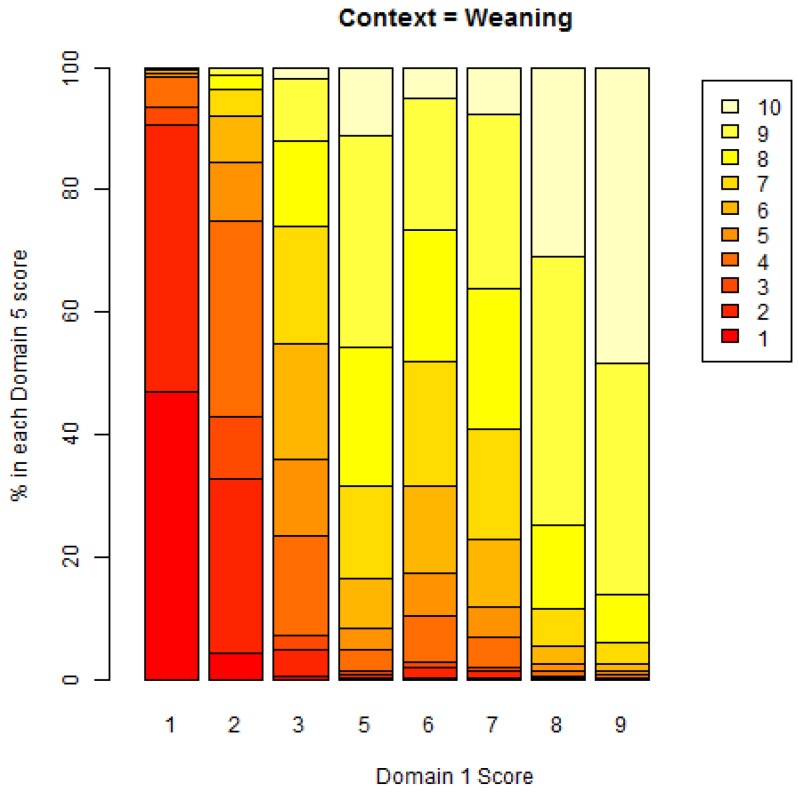
A graphical illustration of the relationships between scores for Domain 1 and Domain 5 for weaning.

A graphical example showing the pattern of association is given for the context of weaning in Figure 17 below. The predictions of the model based on the ordinal GLMM regression output are shown visually. For example, on 50% of occasions, a Domain 1 score of 9 would result in a Domain 5 score of 10.

## 4. Discussions

Rather than the specific scores that the panel applied, the focus of the current discussion is on how we applied the Five Domains Model to assess the adverse impacts of husbandry, veterinary, and equitation interventions on horse welfare. Clearly, the very nature of the interventions considered can have a variable influence on the interrelationships between domains. We accept that the aversiveness scores for many of the procedures considered reflect the panel’s perception of what horses feel, and that there is a need for more study of non-invasive metrics of stress in horses, and their capacity to habituate to aversive events. We acknowledge that commercial practitioners’ perceptions of the impact of the current interventions may differ markedly from those of the current panel.

We also acknowledge the need to consider the balance between the costs and benefits of interventions, and the merits of encompassing the evidence for a positive affective state in future endeavors of this kind. Our discussion includes some commentary on specific interventions and associations between pre-workshop and post-workshop impact scores. It explores how the workshop exposed some potential research gaps and limitations of the current framework, before offering recommendations for the conduct of similar exercises in the future.

### 4.1. Adverse Effects and HBA

Panellists at this workshop considered the negative impact on the welfare of domestic horses of various common interventions within 14 contexts. Only potential harms were considered. It is recognised that some of the interventions will have had a long-term benefit for the horses themselves and/or for the owners. We have not been proscriptive as to what should or should not be done. We have simply attempted to assess, and invite consideration, as to how these things might be experienced by the horse. The panellists did not offer any ethical opinion as to the justifications for procedures that benefit only the owner, except to say that these are questions that merit critical attention.

For nearly all of the procedures that we considered within the care context, such as mane pulling, we ranked the welfare costs as mild (having an overall rank in the workshop Domain 5 of 3 or less, see Supplementary Materials Table S8). For those who believe that the beauty of the horse lies primarily in its function, these interventions may seem pointless, but given their apparently mild impact, they need not be a source of great concern. However, the current workshop did identify a number of procedures within the husbandry, equitation, and veterinary contexts where harm to the horse was either moderate (having an overall rank in the workshop Domain 5 of between 3 and 6) or substantial (having an overall rank in the workshop Domain 5 of more than 6). In these cases, it is proper to examine and seek to justify the potential benefits to the horse and owner. Some of these procedures are summarised in Table 4.

### 4.2. Comments on Specific Interventions

The current assessments must be considered in the light of our assumptions (Supplementary Materials Tables S1–S14), but the nominal scores we have assigned for the five domains may be considered either separately or in combination. For example, abrupt weaning was ranked as a moderate–substantial stress, which would be compounded if the foal were then put into isolation. Weaning is associated with a short-term stress response, loss of a significant conspecific, sleep loss, and the disruption of resting behaviours, but it is also unavoidable in nearly all circumstances. Best practice is based on the recognition of the stress involved and choice of measures taken to minimise it. This may include minimal disruption of the foal’s nonmaternal social milieu to constrain the impact of weaning on Domain 4.

Even though they may provide adequate nutrition, any deviations from forage-based diets were identified as creating a series of both health and behavioural challenges. The health challenges probably arise on a pro-rata basis related to the percentage and the type of concentrate, and include risks of fungal allergens, botulism, contamination from faecal material on the floor, and risks to gastrointestinal health in the form of ulcers, choke, and colic. Meanwhile, the behavioural challenges include not moving while ingesting (because the horse’s natural behaviour is to eat and move concurrently), which has implications for exercise and several consequences that, again, have impacts on a pro-rata basis. These include effects on time budgets, choice, arousal before food delivery, a potential increase in food-related aggression (food-guarding), and a potential for “over-heating” with high-energy concentrates.

Housing a horse in isolation, whether free-standing or tethered, was considered a substantial affront to welfare. In a stable yard with many animals, this should rarely be necessary, except possibly for medical reasons. It was noted that, because security is a powerful motivator, a lone outdoor horse must spend more time being vigilant even when accommodated outdoors, which is an outcome that may affect sleep patterns. It was also noted that many paddocks are relatively barren. For owners with only one horse, it may appear to be unavoidable. However, recognition of the severity of the distress that this can cause should persuade owners to consider more sympathetic housing arrangements.

Branding was deemed necessary in some circumstances. Once again, the aim should be to follow best practice through (in this case) minimising the severity and duration of pain. In this regard, freeze branding is clearly preferable to hot branding [34].

Use of an anti-cribbing collar to prevent crib-biting and wind-sucking was considered to cause mild physical discomfort linked not least to the reduced ability to swallow (Table S8), but more severe psychological distress through frustration of an activity that probably originated as a strategy to relieve abdominal discomfort but may later have developed into a stereotypy [35]. Better attention to diet, especially at weaning, should reduce the risk of the horse developing this behaviour pattern [36].

The use of severely restrictive nosebands for competition horses in order to increase control and reduce oral signs of resistance was considered to cause substantial discomfort. Moreover, it may compromise the normal behaviour of the horse [37], and in some cases, make riders reliant on its use.

Veterinary surgical procedures listed in the table include those essential to survival (e.g., colic surgery), those necessary for management (castration), and those for which there is little or no justification (e.g., clitorectomy). Where the procedure is necessary, the responsibility of the veterinarian should be to ensure best practice. In the case of castration, the group concluded that this should include pre-operative sedation and analgesia, surgery on a standing colt with local anaesthesia, and post-operative analgesia for as long as necessary. It was noted that castration without veterinary supervision brings significant risks to welfare (Supplementary Materials Table S7). With regard to the long-term quality of life for a male horse, castration has obvious pluses and minuses. We suggest, but include among other considerations, that most of the time, in most equine establishments, the welfare of geldings is superior to stallions.

Clitorectomy was originally claimed to reduce the risk of the transmission of contagious endometritis from symptomless mares. The approach was inherently crude, since it was based on the absence of a diagnosis. There is now a good bacteriological test, which should render this procedure redundant [38]. It is anticipated that other interventions considered by this workshop may fall out of favor when there are sufficient data to support an exhaustive HBA.

### 4.3. Potential Research Gaps

The workshop revealed a series of potential research gaps, including any harmful effects of feeding/overfeeding supplements, the need to consider the horse’s ethological needs for space, and various challenges faced by horses in transport, including the potential for motion sickness in horses. Similarly, the panellists also queried the cost–benefit of blinkers to restrict vision in terms of putative advantages for racing/carriage horses versus aversive effects and negative welfare consequences. The common perception that pasture matings are risky should be weighed against the welfare benefits and potential flow-on effects of these benefits to the lives of breeding stallions and mares. It was agreed that further research is needed into the consequences of early weaning, and also of lactating mares being separated from their own foals in order to become wet nurses. The welfare of the biological foal, the transferred foal, and the mother of the transferred foal need to be evaluated.

### 4.4. Pre-Workshop and Post-Workshop Impact Scores

Generally, the pre-workshop scores aligned well with the workshop rankings for Domain 5. The alignment for surgical interventions and breeding mares was less than for other contexts, probably because not all of the panellists were familiar with veterinary interventions and breeding practices.

Some changes between pre-workshop and workshop scores were probably due to the clarification of assumptions, e.g., this may explain why the scores for hot shoeing went down, whereas those for the Hobday procedure went up. Meanwhile, shifts in other scores may reflect the effects of this international workshop regarding the sharing of knowledge in the light of differing experiences, e.g., urine harvesting from pregnant mares is not conducted in the UK. As would be expected, participants assigning lower pre-workshop scores tended to give lower workshop scores for Domain 5. In the light of the workshop discussions among the panellists, Domain 1 showed significant alignment with pre-workshop scores for all contexts apart from diet, elective procedures, and restraint for management, transport, competition, and work (see Table 1). Feeding 100% concentrate was scored as having the greatest adverse impact within this Domain in both pre-workshop and workshop assessments, chiefly because of its adverse effect on behaviour (see Figure 3). So, workshop discussions around the impact of interventions on nutrition may have been particularly influential, and even transformative. In contrast, Domain 2 was aligned with pre-workshop scores for most contexts, apart from medical interventions and restraint procedures, some of which, such as hobbling, seemed more familiar to Australian attendees than those from elsewhere. Similarly, Domain 3 showed significant alignment with pre-workshop scores for all of the contexts, apart from transport, while Domain 4 was significantly aligned with pre-workshop scores for all of the contexts, suggesting that the perceived impact of behavioural restrictions on welfare was predominant and relatively immutable.

### 4.5. Limitations of the Current Framework

There are several limitations to the approach reported here that merit consideration. The interventions that were considered at this workshop were not identified via a structured review of the relevant literature, so their selection for this exercise may reflect the backgrounds, experiences, and biases of the panellists. We acknowledge that there are significant gaps in the evidence for the general assumptions, which we have listed in detail. These limit objective evaluations of interventions using the Five Domains model.

The assumption of best practice and standard practice may be an error, since many people use neither best nor standard practice. We also acknowledge that standard practice varies between countries. In some cases, it only takes one person not to follow best practice to have significantly worse welfare outcomes for horses. In such cases, our assessments will fail to reflect the severity of an intervention. It is also important to acknowledge that the current approach lacks explicit consideration of individual differences. For example, indoor housing may be worse for certain horses than for others, depending on factors including previous learning, personality, and personal preferences.

There may have been systematic biases (or poor inter-assessor reliability) due to not considering cumulative suffering or the modulation of effects. In some cases, different procedures may aggregate (e.g., multiple procedures) or compound other factors, such as those regarding diet, social, stress, and health. Conversely, the effects of one intervention may be reduced by those of another. The panellists at the current workshop assumed that duration has a consistent effect over time (i.e., it has a linear relationship with importance to the animal), but duration may be of different significance (a) for different affective states (e.g., the cumulative effects of hunger versus thirst) or (b) for different animals. This leads to the suggestion that one might consider duration as a percentage of the (remaining) lifespan or as an absolute timespan. By limiting duration (e.g., of care procedures), the panellists ruled out many of the sequelae, which may be more important in some cases than others. The duration of the intervention (and duration as defined in assumptions) may obviously not be the duration of the resultant affective states.

It is also important to note that independence among panellists is likely to have changed over the course of the workshop, not least as a result of some explicit discussions about the intensity and severity of interventions. Where one person had more experience on an issue, there is a risk of others following them. This may be especially important for our assumptions, including those around best practice. A lack of familiarity with a procedure can greatly limit an individual’s ability to provide an assessment, and may have an effect on the perception of the severity or lack of severity of the scores allocated during this workshop. Some of the scores reported reflect this, and could indicate some potential shortfalls across the panel’s experience.

It is likely that time constraints thwarted discussions regarding affective states. The panel did sometimes explicitly note affective states (usually in Domain 4, for example, anticipating the impact of pain and frustration) regarding behavioural processes that imply a judgment regarding affective states (e.g., punishment and habituation). In the current workshop, this may represent a potential bias that reflects the panellists’ professions and experiences. The framework offered no obvious place to include behavioural indicators, so these were usually included in Domain 4. Additionally, it was not explicitly clear how to aggregate scores when assessing impact under Domain 5. It is possible that, when arriving at a score for Domain 5, different panellists may have averaged scores from Domains 1–4, summed them, or simply taken the maximum number. The use of four domains may imply that each is equally important, which may not be the case. In general, it may be that health considerations are more important than behaviour, or vice versa. Furthermore, it could be that, in some cases, only the highest-scoring domain is important (e.g., a catastrophic health issue may make diet irrelevant). This limitation also made it difficult to know in which domain to record interventions that have multiple facets (e.g., social contact may modulate stress). Indeed, Domain 4 (Behaviour) risks becoming a catch-all for any impact (such as oral satisfaction) that is either general or does not fit easily into another domain. Finally, we note that the linear scale we used in the workshop was applied uniformly across all of the interventions that were assessed. However, it is important to emphasize that it was not validated.

### 4.6. Recommendations

In the current workshop, scoring was always conducted *within* contexts. There may be merit in including an additional stage of assessment so that scoring across contexts could be checked. So, for example, future workshops of this sort might include an extra session at the closure of proceedings, in which interventions from different contexts could be considered together, e.g., all of the interventions that had received a Domain 5 score of 5 could have been pooled together and re-ranked. Such a process would help to show whether a score of, say, 7 within one context was the same as a score of 7 within another.

During the current workshop, it was not explicit whether the numbers that appeared on the scoring scales were merely ordinal or were also cardinal (and on which scale). The absence of a zero on the scale means that it was not a ratio scale, but it is possible that some panellists may have assumed so. Indeed, very occasionally (0.07% of responses), zero scores were assigned. The organisers of future workshops of this sort may wish to clarify ahead the scale type they deploy. In the current workshop, the impact of interventions was ranked on a 10-point scale, but some panellists argued that, for practical purposes, it may be sufficient to use the three bands: mild, moderate, and substantial. It was also suggested that the short and long-term consequences should be ranked separately. A validated scale should be selected for further related exercises.

We accept that because we asked panellists simply to assess the welfare impact of interventions on a linear scale before the workshop, the relevance of the resultant pre-workshop scores and the statistical associations with them are difficult to interpret. More objective guidance around any pre-workshop score would add value to it as a comparative variable in future exercises of this sort. Since the panellists had already been exposed to the use of the impact scores before the current workshop, there may have been an element of repeated measures. Ideally, in future iterations of this exercise, a group of panellists who have not seen scores before the workshop could serve as a comparison group to account for this.

Once the preliminary master list of interventions had been compiled, panellists were asked to contribute their collective knowledge and experience to further refine the final list for scoring. This proved to be one of the most illuminating aspects of the workshop, reflecting the rich background from which the panel was drawn. It is anticipated that defining the most common interventions and delineating assumptions upon which welfare assessments can be made will help build a bedrock on which future assessment exercises of this sort can be based. It is worth noting that future workshops could usefully address opportunities or positive factors or states.

## 5. Conclusions

Horse welfare can be estimated using the Five Domains model. The model offers a framework for discussions around the impact within each domain. However, defining the interventions and delineating assumptions upon which the panellists make their assessments is a pivotal workshop activity. In future, the assumptions presented here may lead to a scoring system that allows horse carers to map the impacts of various management, training, and competition activities on horse welfare. Furthermore, the basis of this model can be extended to other domestic and captive species (e.g., working elephants and camels) in order to provide a unique and perhaps more accurate and holistic assessment of welfare than previous approaches.

## Figures and Tables

**Table 1:** *P*-values for associations between Domain 1 to 4 scores, as well as pre-workshop score (Pre WS) and Domain 5 score. Each entry represents a separate ordinal generalised linear mixed model (GLMM).

Context	Domain 1	Domain 2	Domain 3	Domain 4	Pre WS
Weaning	5.4×10^–9^	1.7×10^–8^	8.8×10^–14^	<2.2×10^–16^	7.2×10^–6^
Diet	0.11	*	<2.2×10^–16^	<2.2×10^–16^	1.6×10^–13^
Housing	*	<2.2×10^–16^	<2.2×10^–16^	<2.2×10^–16^	<2.2×10^–16^
Foundation training	*	0.0053	<2.2×10^–16^	<2.2×10^–16^	8.6×10^–5^
Medical interventions	0.011	0.18	1.3×10^–6^	7.6×10^–5^	0.073
Surgical interventions	0.00018	0.031	0.00095	5.6×10^–^0^9^	0.14
Elective procedures	0.24	0.00081	<2.2×10^–16^	<2.2×10^–16^	<2.2×10^–16^
Care procedures	1.8×10^–7^	0.0041	<2.2×10^–16^	<2.2×10^–16^	<2.2×10^–16^
Restraint for management	0.50	0.63	<2.2×10^–16^	<2.2×10^–16^	0.00025
Transport	0.51	0.013	0.22	5.0×10^–8^	3.7×10^–5^
Activity: competition	0.82	8.9×10^–13^	<2.2×10^–16^	<2.2×10^–16^	0.0030
Activity: work	0.61	6.5×10^–7^	1.2×10^–6^	<2.2×10^–16^	0.028
Activity: breeding mares	0.00052	2.4×10^–7^	0.025	7.5×10^–11^	0.73
Activity: breeding stallions	*	0.0038	0.00059	4.1×10^–11^	0.00068

* Association test not possible as all Domain scores were equal.

**Table 2:** Regression coefficients and standard errors for associations between pre-workshop scores and Domain 5 scores assigned to assess the adverse impacts of husbandry, veterinary, and equitation interventions on horse welfare across 14 contexts: Weaning; Diet; Housing; Foundation training; Ill-health and veterinary interventions (chiefly medical); Ill-health and veterinary interventions (chiefly surgical); Elective procedures; Care procedures; Restraint for management procedures; Road transport; Activity—competition; Activity—work; Activity—breeding females; and Activity—breeding males.

	**Weaning**		**Diet**		**Housing**		**Foundation training**		**Medical**		**Surgical**		**Elective Procedures**	
Pre-workshop														
Score	Estimate	Std. Error	Estimate	Std. Error	Estimate	Std. Error	Estimate	Std. Error	Estimate	Std. Error	Estimate	Std. Error	Estimate	Std. Error
1	0.00		0.00		0.00		0.00						0.00	
2	−1.62	1.30	−3.77	1.26	2.97	1.05	2.84	1.26	0.00		0.00		−4.20	0.60
3	−0.79	1.26	−2.54	1.12	3.22	1.05	3.64	1.48	−0.88	NA	2.66	2.18	−1.18	0.58
4	0.27	1.11	−1.00	1.13	4.08	1.05	5.31	1.69	−2.30	NA	0.00	2.23	−0.92	0.66
5	1.37	1.14	0.43	1.18	4.05	1.05	5.03	1.51			4.49	2.31	−0.95	0.50
6	2.28	1.15	−0.27	1.34	5.13	1.07	4.60	1.45	−2.67	NA	3.46	2.12	−1.00	0.52
7	3.35	1.30	1.43	1.08	6.13	1.05	6.65	1.75	2.12	NA	1.28	2.90	0.39	0.64
8	2.89	1.18	3.02	1.15	6.97	1.08	6.83	1.89			5.88	2.47	0.72	0.54
9	3.35	1.43	4.57	1.34	8.32	1.11	6.79	2.39			5.11	2.34	1.74	0.63
10	6.38	1.73	4.45	1.44	9.42	1.13	7.16	2.01			5.66	2.57	3.98	0.62
	**Care Procedures**		**Restraint**		**Transport**		**Competition**		**Work**		**Breeding Females**		**Breeding Males**	
Pre-workshop														
Score	Estimate	Std. Error	Estimate	Std. Error	Estimate	Std. Error	Estimate	Std. Error	Estimate	Std. Error	Estimate	Std. Error	Estimate	Std. Error
1	0.00		0.00				0.00		0.00		0.00		0.00	
2	−0.33	0.49	1.54	0.94	0.00				−1.60	NA	0.22	NA	−27.59	NA
3	0.11	0.46	1.42	1.00	−23.58	NA	−3.27	1.42	−3.93	NA	−0.74	NA	−22.47	NA
4	0.64	0.54	3.04	1.06	−1.42	NA	0.64	1.14	−1.36	NA	0.32	NA	−19.45	NA
5	0.84	0.55	2.76	1.06	−3.46	NA	0.41	1.09	−0.84	NA	−0.80	NA	−26.36	NA
6	2.33	0.68	3.20	1.15	19.30	NA	1.24	1.05	−0.68	NA	−0.30	NA		
7	2.12	0.61	3.42	1.03	41.07	NA	2.06	1.15	−0.05	NA	2.93	NA		
8	2.86	0.64	3.01	1.08	−20.46	NA	2.69	1.16	0.47	NA			0.00	NA
9	3.38	0.68	3.99	1.20	20.78	NA	2.35	1.38	4.07	NA	0.18	NA		
10	6.01	0.92	5.35	1.37	38.25	NA			2.59	NA			21.40	NA

**Table 3:** Regression coefficients and standard errors for associations between scores for Domains 1–4 and Domain 5 scores.

**Context = Weaning**								
Score in	Domain 1		Domain 2		Domain 3		Domain 4	
Domain	Estimate	Std. Error	Estimate	Std. Error	Estimate	Std. Error	Estimate	Std. Error
1	0.00		0.00		0.00		0.00	
2	2.97	1.38	0.02	1.01	20.36	357.70	9.48	33.06
3	5.24	1.55	2.76	1.07	22.63	357.70	26.12	41.03
4			1.94	1.07	25.36	357.70	26.11	41.02
5	7.06	1.59	4.32	1.15	26.09	357.71	29.66	41.04
6	6.21	1.52	5.08	1.26	27.36	357.71	34.09	41.06
7	6.66	1.51	5.43	1.18	29.65	357.71	34.37	41.06
8	8.32	1.81	6.15	1.68	30.01	357.71	36.60	41.07
9	9.05	1.71	6.76	1.52	32.07	357.72	36.17	41.08
10			25.90	593.16	33.14	357.72	38.10	41.08
**Context = Diet**								
Score in	Domain 1		Domain 2		Domain 3		Domain 4	
Domain	Estimate	Std. Error	Estimate	Std. Error	Estimate	Std. Error	Estimate	Std. Error
1	0.00				0.00		0.00	
2	−0.53	0.43			20.44	105.69	4.33	1.22
3	−0.29	0.52			22.23	105.69	7.95	1.52
4	0.22	0.57			24.80	105.69	9.41	1.61
5	0.26	0.67			26.71	105.69	11.45	1.68
6	0.81	0.75			27.71	105.69	14.57	1.89
7	2.43	1.00			28.51	105.69	14.95	2.01
8					31.54	105.69	18.23	2.06
9					33.12	105.71	21.06	2.34
10								
**Context = Housing**								
Score in	Domain 1		Domain 2		Domain 3		Domain 4	
Domain	Estimate	Std. Error	Estimate	Std. Error	Estimate	Std. Error	Estimate	Std. Error
1			0.00		0.00		0.00	
2			1.27	0.79	−1.02	0.82	3.28	1.41
3			1.55	0.68	0.18	0.78	5.79	1.50
4			1.76	0.70	0.24	0.83	8.27	1.56
5			3.67	0.70	1.84	0.82	9.28	1.57
6			3.73	0.77	2.12	0.88	11.10	1.61
7			5.64	0.81	3.03	0.90	12.79	1.63
8			7.07	0.85	4.75	0.94	15.76	1.71
9			7.98	1.03	5.55	1.16	19.13	1.91
10			8.33	1.30	5.48	1.86	21.64	2.21

**Table 4:** Examples of harm:benefit analysis for elective procedures, drawing from the current panel’s discussions at the workshop.

Procedure	Harm	Benefit to Horse	Benefit to Owner
*Husbandry*			
Housing in isolation	Substantial	None	Sometimes necessary
Hot branding	Moderate	None	Sometimes necessary
Anti-cribbing collar	Moderate	Doubtful	Avoidable
Abrupt weaning (all forms that involve forced physical separation of the foal and dam)	Moderate–substantial	None	Avoidable
*Equitation*			
Restrictive noseband	Moderate	None	Arguable
Tongue ties	Moderate	None	Arguable
*Veterinary*			
Colic surgery	Substantial	Substantial	Substantial
Castration: best practice	Moderate	Probable?	Substantial
Castration: no anaesthetic	Substantial	Probable?	Substantial
Clitorectomy	Moderate	None	Unnecessary

## Supplementary Materials

Tables S1–S14; the workshop handbook including an overview of the context, and annotated references, is available at: www.mdpi.com/2076-2615/8/3/41/s1.

## Appendix A.

**Table A1:** Eliminations (*n* = 60) from pre-workshop list of interventions with brief reasons for deletions.

Context	Intervention Deleted	Reason
Housing	Indoor stable—No social contact with turn-out to paddock	Deleted due to lack of time
	Indoor stable—Partial social contact with turn-out to paddock	
	Indoor stable—Full social contact with turn-out to paddock	
Surgical	Surgical deep intra-cavity surgery (e.g., synovial sepsis, sinusitis)	>Lack of time
Elective procedures	Measuring	Considered likely to be of negligible impact
	Weighing	
	Docking	Banned in most jurisdictions
Care procedures	Hogging	Lack of time or considered likely to be of negligible impact
	Trim outside edge and/or inside ears	
	Apply make-up to eyes, muzzle	
	Apply earplugs	
	Apply nostril applications to change smells	
	Mascara on eyelashes	
	Hosing down	
	Sponging and ice buckets	
	Overnight treatments (e.g., ice bandages etc.)	
	Braiding	
	Mane hogging	
	Scissors to tidy the dock/thin the mane	
	Trimming tails short (level with hocks)	
	Fitting false plaits to manes and tails	
	Washing with non-animal products (e.g., bleach, laundry whitener)	
	Use of different types of brushes	
	Wiping eyes and nostrils with a cloth and dock	
	Coat techniques (e.g., quarter marks, sharks teeth)	
	Clipping	
	Trim chestnuts	
	Coat cleaning/de-tangling products	
	Bot fly knife use	
	Stable bandages to prevent leg-filling/keep clean	
	Use of other boots in stables (e.g., hocks, sausage boot)	
	Care of wet, hairy horses after riding	
	Cleaning out hooves	
	Hoof-shaping for performance traits (e.g., extra-long toes)	
	Shoeing—performance purpose (e.g., weighted hackneys)	
	Trim coronet band	
	Cut off feathers	
	Sandpapering hooves	
	Application of tail raisers and/or irritants to raise the tail	Banned in most jurisdictions
	Performance enhancing irritants (e.g., WD-40 on coronets)	
Restraint	Gag	Too broad
	Hobbles	
	Fencing	
Activity—competition	Showing—training	Lack of time and no benefit in differentiating between training and competition (training considered as part of competition)
	Eventing—training	
	Dressage—training	
	Endurance—training	
	Trail riding—training	
	Western performance (reining)—training	
	Pony club—training	
	Adult riding club—training	
	Driving (Carriage)—training	
	Driving (Carriage)—competition	
	Flat racing—training	
	Jumps racing—training	
	Harness racing—training	
	Polo—training	
Activity—work	Horses in research	Too broad
Activity—breeding females	Donor mare for oocyte transfer/embryo transfer	Lack of time
	Recipient mare for oocyte transfer/embryo	

**Table A2:** The pre-workshop list of original interventions (*n* = 27) that were modified to maximise specificity during the workshop.

Context	Original Intervention	Modified Intervention
Diet	Pasture	Pasture—no choice
		Pasture—choice
Foundation training	Bit habituation	Bit habituation with reins
	Backing	Backing (<18 months)
		Backing (>18 months)
	Side-reining	Side-reining (forced flexion)
Medical	Medical—immediately curative (e.g., medical colic)	Medical—one-off, immediately curative (e.g., spasmodic colic)
	Medical—short-term curative (e.g., sweet itch, uveitis, lameness, kick injury to limb)	Medical—repeated, short-term curative (e.g., fresh superficial mid-cannon injury to a forelimb)
	Medical—long-term curative (e.g., equine gastric ulcer syndrome, equine herpes virus, equine influenza, colitis, diarrhoea, hyperlipaemia, minor tendon injuries, weight loss, laminitis)	Medical—repeated, long-term curative (e.g., equine gastric ulcer syndrome, equine herpes virus, equine influenza, colitis, diarrhoea, hyperlipaemia, minor tendon injuries, weight loss, laminitis)
	Medical—prolonged palliative treatment (e.g., chronic obstructive pulmonary disease (recurrent airway obstruction)	Medical—prolonged palliative treatment (e.g., sweet itch/Queensland itch, chronic obstructive pulmonary disease (recurrent airway obstruction)
Surgical	Surgical single curative intra-cavity surgery or repeated minor surgery (e.g., cryptorchid, osteochondrosis dissecans lesions, osteochondrosis, developmental osteochondral fragments)	Surgical single curative intra-cavity surgery or repeated minor surgery (e.g., inguinal cryptorchid, osteochondrosis dissecans lesions (osteochondrosis,)
-	Surgical major deep intra-cavity surgery (e.g., colic—surgical torsion)	Surgical major deep intra-cavity surgery (e.g., colic—surgical torsion—small intestine resection, developmental osteochondral fragment removal)
Weaning	Weaning with unrelated adults	Weaning with unrelated adults (mares being removed at intervals from an established group of mares and foals)
Care procedures	Neck rug	Rugging
	Hoods	Hoods (applying)
	Collars for windsuckers/crib-biters (or other devices for behaviour modifications)	Collars for windsuckers/crib-biters
	Trimming	Trimming hooves
	Shoeing—general	Shoeing—cold
		Shoeing—hot
		Deworming
Restraint	Headcollar, bridle, bit (of varying levels of severity)	Chifney
		Chain on gums
Road transport	Group—with familiar companions	Group—with familiar companions—individually penned
	Group—with unfamiliar companions	Group—with unfamiliar companions—individually penned
		Group—with familiar companions, penned as a group
Activity—competition	Showing—competition	Showing—in-hand
		Showing—ridden
	Pony club—competition	Community club—competition
	Adult riding club—competition	
		Show jumping
	Polo/polocrosse	Polo
Activity—work	Riding school	Riding school/riding for the disabled/therapeutic riding horse
	Riding for the disabled	
	Therapeutic riding horse	
		Rental stables
Activity—breeding females	-	Brood mare—pasture matings
		Brood mare—in-hand matings
		Brood mare—artificial insemination
Activity—breeding males	Breeding stallion—in-hand matings (including semen collection)	Breeding stallion—in-hand matings
		Breeding stallion—artificial insemination
