# Supplementary material for: Using the Five Domains Model to Assess the Adverse Impacts of Husbandry, Veterinary, and Equitation Interventions on Horse Welfare
Source: Animals (Basel). 2018 Mar 18;8(3):41. doi: 10.3390/ani8030041 (PMC5867529; doi:10.3390/ani8030041)
Supplement: Supplementary file 1 [file animals-08-00041-s001.pdf]

**Supplementary Materials -** Assumptions (shaded text) and notes (unshaded text) made when panellists considered interventions. NB. In some of the Notes in the following Tables, the larger numerals in Column 1 refer to the original pre-workshop categories, hence the apparent gaps.

**Table S1.** Weaning. Assumptions and notes made when panellists considered interventions related to weaning.

| **Duration** | Assessed for the first 72 hours. |
|---|---|

| | **General Assumptions** |
|---|---|
| a | Enclosure is safe; adequate space, flooring and bedding are provided. |
| b | Age at weaning is 4-6 months. |
| c | *Ad libitum* access to familiar forage and water. |
| d | Supplementary feed introduced prior to weaning (*see* p.265 *Horse Sense*). |
| e | Welfare of foal (rather than mare) is being assessed. |

| | **Specific assumptions and Notes** | |
|---|---|---|
| **1** | Abrupt – Individual: Mare out of sight and sound from foal. Prior to weaning, mare and foal are turned out on pasture. The foal has been abruptly moved into an unfamiliar standard-size fully enclosed stable on its own for 24 hours.<br>**DOMAIN** | |
| | **1** | Risk of inappropriate diet if high-concentrate feed given. Abrupt removal of mare's milk but foals over 4 months of age are unlikely to be dependent on milk as their chief source of nutrition. |
| | **2** | Unfamiliar, barren environment that may impose atmospheric pollutants, restricted and or constant light, and restrict locomotory activity. |
| | **3** | Risk of digestive disorder or pathology. Significant risk of injury and weight loss. |
| | **4** | Short-term stress response, social isolation and loss of significant conspecific, sleep loss and resting behaviours affected. Increased risk of oral and locomotory stereotypies. |
| **2** | Abrupt – Paired: A familiar pairing, in an unfamiliar standard-size stable fully enclosed for 24 hours.<br>**DOMAIN** | |
| | **1** | Risk of inappropriate diet if high-concentrate feed given. Abrupt removal of mare's milk but foals over 4 months of age are unlikely to be dependent on milk as their chief source of nutrition. |
| | **2** | Unfamiliar, barren environment, atmospheric pollutants, restricted and or constant light, movement restrictions. Reduced space available per foal compared with individual weaning in a stable of the same size. |
| | **3** | Risk for socially induced injury increased, relative to one foal in the stable. Risk of digestive disorder or pathology. Significant risk of injury and weight loss. |
| | **4** | Short-term stress response, loss of significant conspecific, sleep loss and resting behaviours affected. Risk of oral stereotypies. May attempt sucking behaviour. |
| **3** | Abrupt – Group: A familiar group, in an unfamiliar secure barn. Stocking density no greater than paired foals. Forage appropriately distributed.<br>**DOMAIN** | |
| | **1** | Risk of inappropriate diet if high-concentrate feed given. Abrupt removal of mare's milk but foals over 4 months of age are unlikely to be dependent on milk as their chief source of nutrition. |

| | | |
|---|---|---|
| | **2** | Unfamiliar environment, atmospheric pollutants, restricted and or constant light, fewer movement restrictions compared to stable. |
| | **3** | Risk for socially induced injury is higher, relative to one or paired foals in the stable. Risk of panic-induced injury is lower. Risk of digestive disorder or pathology. Significant risk of weight loss. |
| | **4** | Short-term stress response, loss of significant conspecific, sleep loss and resting behaviours affected. Risk of oral stereotypies. May attempt sucking behaviour. More opportunities for herd behaviours. |
| **4** | Partial separation: Permanent fence placed between mare and foal, where fence does not permit suckling. Foal familiar with enclosed area. No physical contact other than head to head. On pasture. Single foal.<br>**DOMAIN** | |
| | **1** | Risk of inappropriate diet if high-concentrate feed given. Abrupt removal of mare's milk but foals over 4 months of age are unlikely to be dependent on milk as their chief source of nutrition. |
| | **2** | – |
| | **3** | Risk of panic-induced injury. Risk of digestive disorder or pathology. Risk of weight loss. |
| | **4** | Short-term stress response, loss of significant conspecific, sleep loss and resting behaviours affected. Risk of oral stereotypies. Isolation from herd. |
| **5** | Weaning with unrelated adults: Left with familiar herd foals. In a familiar pasture environment with the presence of a "nanny" horse (unrelated familiar non-lactating mare or gelding). Assessment focussed on the welfare of the foal of first mare removed from group.<br>**DOMAIN** | |
| | **1** | Risk of inappropriate diet if high-concentrate feed given. Abrupt removal of mare's milk but foals over 4 months of age are unlikely to be dependent on milk as their chief source of nutrition. |
| | **2** | – |
| | **3** | Risk for socially induced injury is lower, relative to one or paired foals in the stable or group weaning. Risk of panic-induced injury is lower than for partial separation. Risk of digestive disorder or pathology. Risk of weight loss. |
| | **4** | Short-term stress response, loss of significant "other", sleep loss and resting behaviours affected. Risk of oral stereotypies lower. May attempt sucking behaviour. More opportunities for herd behaviours. |
| **6** | Natural: Mares and foals at pasture. Mare and foal stay in the same herd until the mare has another foal. Foal continues to suckle for 12-18 months.<br>**DOMAIN** | |
| | **1** | – |
| | **2** | – |
| | **3** | Risk of panic-induced injury and socially induced injury is low, relative to one or paired foals in the stable, group or unrelated adult weaning. Risks of digestive disorder or pathology and weight loss is low relative to all other methods. |
| | **4** | Not entirely stress-free. |

**Table S2.** Assumptions and notes made when panellists considered interventions related to diet.

| | |
|---|---|
| **Duration** | Daily feeding for 1 year. |

Pre-workshop assumptions: Good quality pasture; appropriate micronutrients; established diet; access to diet without bullying; *ad libitum* water

| | **Assumptions** |
|---|---|
| a | Well-managed pasture suitable for horses. |
| b | Acknowledge seasonal change in body condition and potential change in appetite linked with feed availability. |
| c | Parasite control through rotational grazing, cross grazing. |
| d | Appropriate nutrients, including no micronutrient deficiency. |
| e | Established diet (not in transition from one diet to another). |
| f | Access to food without bullying. |
| g | *Ad libitum* water provided. |
| h | No supplements. |
| i | Best practice parasite control (of horse rather than pasture). |
| j | Same assumptions for exercise/status of horse as per housing (healthy horse with healthy teeth). |
| k | Assume fencing/housing is safe. |
| l | Effective preventative medicine program. |
| | **For concentrates and cut forage:** Assume fed from a bucket/bin on the floor; no access to other food sources; fed 2 times/day; energy chiefly from carbohydrate source. Assume energy density of concentrate should be dependent on activity level of horse. |
| | **For cut forage:** Rationed (i.e., periods without access to forage); long-stemmed fibre; reliable supply from same source; correctly stored and free of toxic plants; assume no need to soak. |
| | **For concentrate only:** Short fibres; fed 5 times/day. |
| | **Donkeys:** Grass is not suitable; require browsing opportunities. |
| | **Note:** (include below where necessary) Risk of increased incidence and prevalence of oral stereotypies with low proportion of forage and/or high proportion of high-carb diet. |

| | | **Notes** |
|---|---|---|
| **1A** | Pasture only – no choice.<br>**DOMAIN** | |
| | **1** | Risk of overeating.<br>No opportunity to choose drier food. |
| | **2** | *Impact assessed separately under housing.* |
| | **3** | Risk of metabolic dysfunction (e.g., obesity/laminitis).<br>Risk of sand ingestion in some environments.<br>Risk of fungal toxicity in monocultures in some environments. |
| | **4** | Motivation for fibre consumption is likely motivation for gut satisfaction.<br>May learn which substrate is associated with latent gut satisfaction.<br>Restricted choice. |
| **1B** | Pasture only – with choice (i.e., varied pasture including browse).<br>**DOMAIN** | |
| | **1** | Risk of overeating. |
| | **2** | *Impact assessed separately under housing.* |
| | **3** | Low risk of toxicity from browse. |

| | | |
|---|---|---|
| | | Low injury, laceration risk from browse (e.g., thorns). |
| | **4** | Greater choice and variety/novelty.<br>Movement and postural variation for accessing food. |
| **2** | Cut forage (limited choice).<br>**DOMAIN** | |
| | **1** | Adequate nutrition. |
| | **2** | *Impact assessed separately under housing.* |
| | **3** | Risk of fungal allergens<br>Risk of contamination from floor.<br>Risk of botulism.<br>Not moving while ingesting (fed at one point) – implications for gut health. |
| | **4** | Fibre length is important.<br>Not moving while ingesting (fed at one point) – implications for exercise.<br>Providing straw and forage mix and spreading food around will reduce periods without forage.<br>Time budget.<br>More restrictions on choice to varied pasture.<br>Reduction in seasonal variation compared to varied pasture.<br>Potential risk of frustration before delivery of forage. |
| **3** | 20% Concentrate:80% Forage – high-energy processed concentrate.<br>**DOMAIN** | |
| | **1** | Adequate nutrition. |
| | **2** | *Impact assessed separately under housing.* |
| | **3** | Not moving while ingesting (fed at one point) – implications for gut health.<br>**Pro-rata related to % and type of concentrate:**<br>Risk of fungal allergens.<br>Risk of botulism.<br>Effect of food composition on dentition (changes how horses chew).<br>Risk of contamination from floor.<br>Risk to GI health from diet (ulcers, choke, colic). |
| | **4** | Not moving while ingesting (fed at one point) – implications for exercise<br>**Pro-rata related to % and type of concentrate:**<br>Time budget<br>Slight element of choice<br>Some variation in diet.<br>Arousal before delivery.<br>Potential increase in food-related aggression (food-guarding).<br>Potential for “over-heating” with high-energy concentrates. |
| **4** | 20% Concentrate:80% Forage – low-energy concentrate.<br>**DOMAIN** | |
| | **1** | Adequate nutrition. |
| | **2** | *Impact assessed separately under housing.* |
| | **3** | Not moving while ingesting (fed at one point) – implications for gut health.<br>**Pro-rata related to % and type of concentrate:**<br>isk of fungal allergens.<br>isk of botulism.<br>ffect of food composition on dentition (changes how horses chew). |

| | | |
|---|---|---|
| | | isk of contamination from floor.<br>isk to GI health from diet (ulcers, choke, colic). |
| | **4** | Not moving while ingesting (fed at one point) – implications for exercise.<br>**Pro-rata related to % and type of concentrate:**<br>Time budget.<br>Slight element of choice.<br>Some variation in diet.<br>Arousal before delivery.<br>Potential increase in food-related aggression (food-guarding).<br>Potential for "over-heating" with high-energy concentrates. |
| **5** | 80% Concentrate:20% Forage – high-energy concentrate.<br>**DOMAIN** | |
| | **1** | Adequate nutrition. |
| | **2** | *Impact assessed separately under housing.* |
| | **3** | Not moving while ingesting (fed at one point) – implications for gut health.<br>**Pro-rata related to % and type of concentrate:**<br>Risk of fungal allergens.<br>Risk of botulism.<br>Effect of food composition on dentition (changes how horses chew).<br>Risk of contamination from floor.<br>Risk to GI health from diet (ulcers, choke, colic). |
| | **4** | Not moving while ingesting (fed at one point) – implications for exercise.<br>**Pro-rata related to % and type of concentrate:**<br>Time budget.<br>Slight element of choice.<br>Some variation in diet.<br>Arousal before delivery.<br>Potential increase in food-related aggression (food-guarding).<br>Potential for "over-heating" with high-energy concentrates. |
| **6** | 80% Concentrate:20% Forage – low-energy concentrate.<br>**DOMAIN** | |
| | **1** | Adequate nutrition. |
| | **2** | *Impact assessed separately under housing.* |
| | **3** | Not moving while ingesting (fed at one point) – implications for gut health<br>**Pro-rata related to % and type of concentrate:**<br>Risk of fungal allergens.<br>Risk of botulism.<br>Effect of food composition on dentition (changes how horses chew).<br>Risk of contamination from floor.<br>Risk to GI health from diet (ulcers, choke, colic). |
| | **4** | Not moving while ingesting (fed at one point) – implications for exercise.<br>**Pro-rata related to % and type of concentrate:**<br>ime budget.<br>ight element of choice.<br>ome variation in diet.<br>rousal before delivery.<br>otential increase in food-related aggression (food-guarding).<br>otential for "over-heating" with high-energy concentrates. |

| | | |
|---|---|---|
| **7** | 100% Low-energy processed diet (marketed as a “complete” diet).<br>**Note**: many examples of horses from across panel of horses on 100% concentrate diet.<br>**DOMAIN** | |
| | **1** | Adequate nutrition. |
| | **2** | *Impact assessed separately under housing.* |
| | **3** | Effect of food composition on dentition (changes how horses chew).<br>Risk of contamination from floor.<br>Risk to GI health from diet (ulcers, choke, colic).<br>Not moving while ingesting (fed at one point) – implications for gut health. |
| | **4** | Not moving while ingesting (fed at one point) – implications for exercise.<br>Time budget.<br>No choice.<br>No variation in diet.<br>Arousal before delivery.<br>Potential increase in food-related aggression (food-guarding). |

**Table S3.** Assumptions and notes made when the panellists considered interventions related to housing.

| | |
|---|---|
| **Duration** | Continuous for 1 year. |

Pre-workshop assumptions: Bedding (non-edible) is provided; *Ad libitum* water and no impact as a result of diet (dealt with under dietary manipulation); Shelter from wind, flies and precipitation is provided; Any conspecifics are part of an established social group; No automatic/operant feeders are being used; No non-feeding environmental enrichment; The enclosure is safe with well-maintained appropriate fencing; Stocking density is adequate for all horses to lie and rise safely.

| | **Assumptions** |
|---|---|
| a | Bedding (non-edible) is provided. |
| b | *Ad libitum* water is provided (except for outdoor tethering). |
| c | No impact as a result of diet (diet is dealt with under dietary manipulation). |
| d | Any conspecifics are part of an established social group. |
| e | Shelter from wind, flies and precipitation is provided (except for outdoor tethering). |
| f | No non-feeding environmental enrichment is provided. |
| g | No automatic/operant feeders are being used. |
| h | The enclosure is safe with well-maintained, appropriate fencing. |
| i | Stocking density is adequate for all horses to lie in lateral recumbency and rise safely. |
| j | Where yards are considered, horses can access them freely but they offer only limited opportunities for locomotion (i.e., the yards are small (e.g., 10m x 10m)). |
| k | Horses are adult, healthy and have opportunity for ridden work/exercise for 1 hour/day. |
| | **For partial social contact**: Assuming that at least one other horse is in visible close proximity and that tactile contact is available (limited mutual grooming); companion horse leaves for 1 hour of work or exercise. |
| | **For full social contact**: One companion only; companion horse leaves for ridden work/exercise. Composition of groups is also important. Assuming that this horse is in a group and is able to fully interact with at least one other horse. |

<table>
<tr><th></th><th colspan="2">Notes</th></tr>
<tr><td rowspan="5">1</td><td colspan="2">Outdoor – No social contact.<br>Note: If social isolation increases risk of aggression/stereotypies then this may affect management.<br>DOMAIN</td></tr>
<tr><td>1</td><td>Impact assessed separately under diet.</td></tr>
<tr><td>2</td><td>Opportunity for rotation of paddock will improve outcome.<br>Many horses adaptable/tolerant of very low temperatures.<br>Erosion of paddock due to repetitive tracking.<br>Dust may be an issue in dry climate/mud in wet.<br>Ice may affect drinking behaviour.<br>No opportunity to go indoors.</td></tr>
<tr><td>3</td><td>Risk of fencing injuries (low but not zero).<br>No risk of injuries from conspecifics.<br>Mud fever – linked to wet legs/contact with mud.</td></tr>
<tr><td>4</td><td>Constraints on horse-horse interactions; lack of tactile contact, fly prevention, grooming.<br>Increase in flightiness in response to perceived threats (especially for younger horses; unfamiliar environments). Horses will work for access to a paddock with or without social contact.<br>Horses will work for social contact and will work harder to access companionship (direct or over a barrier) than for an empty paddock. This need is not satisfied.<br>Isolated horses show more behavioural indicators of distress than horses in groups but physiological indicators may be contradictory and may depend on horse age and familiarity with environment.<br>Many paddocks are relatively barren.<br>Security is a powerful motivating factor – lone outdoor horse must spend more time being vigilant; may affect sleep patterns.</td></tr>
<tr><td rowspan="5">2</td><td colspan="2">Outdoor – Partial social contact.<br>DOMAIN</td></tr>
<tr><td>1</td><td>Impact assessed separately under diet.</td></tr>
<tr><td>2</td><td>–</td></tr>
<tr><td>3</td><td>Marginal increase in risk of injury from conspecific.<br>Higher risk of infectious disease through direct contact with conspecific.</td></tr>
<tr><td>4</td><td>Robust demand for partial social contact is satisfied.<br>No behavioural indicators of distress if horses are compatible.<br>Potential for acute separation is greater if outdoor areas are large as companion horse can move a considerable distance away (in contrast to horses isolated indoors).<br>Companion horse may/may not be compatible – size does matter (e.g., for optimal allo-grooming) (Shetland vs Shire).<br>Sleep may be better because of the reduced need for vigilance.<br>Slightly reduced fear of predation.</td></tr>
<tr><td rowspan="4">3</td><td colspan="2">Outdoor – Full social contact.<br>DOMAIN</td></tr>
<tr><td>1</td><td>Impact assessed separately under diet.</td></tr>
<tr><td>2</td><td>–</td></tr>
<tr><td>3</td><td>Increased risk of parasite infection.<br>Slight increased risk of infectious disease.<br>Nic – reference to disease risk reducing in groups.</td></tr>
</table>

| | | |
|---|---|---|
| | | Injury risk in established groups is low. There may be positive outcomes (e.g., opportunities to play). |
| | **4** | Robust demand for full social contact is satisfied.<br>No behavioural indicators of distress if horses are compatible.<br>Physical contact increased with opportunities for allo-grooming and other forms of tactile contact) and reduced fear of perceived threats. Relative size of horses matters for allo-grooming. |
| **4** | Indoor stable – No social contact.<br>**Assumptions:** Standard stable size 3.6mx3.6m (*see Horse Sense*); assume 15h horse; Adequate head clearance. Not a barn environment.<br>**DOMAIN** | |
| | **1** | *Impact assessed separately under diet.* |
| | **2** | Restrictions on physical comfort.<br>Dustier, darker than outdoor.<br>Barren environment. |
| | **3** | Risk of being cast.<br>Increased risk of respiratory disease compared with outdoor housing.<br>Lack of exercise. |
| | **4** | Farm-animal research indicates very high demand for increased space where space is restricted to the point where basic behavioural needs are denied (e.g., turning around and lying down in lateral recumbency).<br>Periods of time when no forage is available/no feeding behaviour.<br>**Note:** If social isolation increases, risk of aggression/stereotypies, then these behavioural outcomes may affect management. |
| **5** | Indoor stable – Partial social contact.<br>**Assumptions:** One horse in close visual proximity; access through a grille.<br>**DOMAIN** | |
| | **1** | *Impact assessed separately under diet.* |
| | **2** | As for "Indoor stable – No social contact". |
| | **3** | As for "Indoor stable – No social contact". |
| | **4** | Robust demand for partial social contact is satisfied.<br>No behavioural indicators of distress if horses are compatible.<br>More social behaviour than for "Indoor stable – No social contact".<br>Separation during companion exercise may cause distress for 1 hour/day. |
| **6** | Indoor stable – Full social contact.<br>**Assumptions:** Pair housing in a stable twice the size of a single stable; horses are compatible.<br>**DOMAIN** | |
| | **1** | *Impact assessed separately under diet.* |
| | **2** | As for "Indoor stable – No social contact". |
| | **3** | As for "Indoor stable – No social contact". |
| | **4** | More social behaviour than for "Indoor stable – Partial social contact". Separation during companion exercise for 1 hour/day. |
| **7** | Indoor tie stall – No social contact.<br>(e.g., Like Army tie stalls, in a barn environment but without social contact.)<br>**DOMAIN** | |
| | **1** | *Impact assessed separately under diet.* |
| | **2** | No turning around; no view of outside world. |

| | | |
|---|---|---|
| | | Restricted ability to self-groom.<br>Air hygiene worse than stable as no direct access to outdoors. |
| | **3** | Restricted muscular activity. |
| | **4** | Fixed eating height. Same concerns for DOMAIN 4 as in “Indoor stable – No social contact”. |
| **8** | Indoor tie stall – Partial social contact.<br>**Assumptions:** Normal Army-style barn with stalls.<br>**DOMAIN** | |
| | **1** | *Impact assessed separately under diet.* |
| | **2** | As for “Indoor tie stall – No social contact”. |
| | **3** | As for “Indoor tie stall – No social contact”. |
| | **4** | Social concerns same as for “Indoor stable – Partial contact”. |
| **9** | Indoor stable – No social contact with free yard access.<br>**DOMAIN** | |
| | **1** | *Impact assessed separately under diet.* |
| | **2** | More choice of ambient temperature than when confined to a stable. |
| | **3** | Fewer risks to health than “Indoor stable – No social contact”. |
| | **4** | Capacity to choose to occupy stable or yard. Same concerns for DOMAIN 4 as in “Indoor stable – No social contact”. |
| **10** | Indoor stable – Partial social contact with free yard access.<br>**DOMAIN** | |
| | **1** | *Impact assessed separately under diet.* |
| | **2** | More choice of ambient temperature than in when confined to a stable. |
| | **3** | Fewer risks to health than “Indoor stable – No social contact”. |
| | **4** | Minimal tactile contact with neighbour. Capacity to choose to occupy stable or yard. Same concerns for DOMAIN 4 as in “Indoor stable – Partial contact”. |
| **11** | Indoor stable – Full social contact with free yard access.<br>**Assumptions:** Pair housing in a stable and yard twice size of single stable/yard; compatible horses.<br>**DOMAIN** | |
| | **1** | *Impact assessed separately under diet.* |
| | **2** | More choice of ambient temperature than when confined to a stable. |
| | **3** | Fewer risks to health than “Indoor stable – No social contact” but more risk of injury than in “Indoor stable – Partial social contact with free yard access”. |
| | **4** | Most demand for social contact is satisfied.<br>No behavioural indicators of distress if horses are compatible. Separation during companion exercise may cause distress for 1 hour/day.<br>Physical contact increased with opportunities for allo-grooming and other forms of tactile contact) and reduced fear of perceived threats. Relative size of horses matters for allo-grooming. |
| **15** | Outdoor tethering – No social contact, no shelter.<br>**Assumptions:** Best standard practice is broad leather collar; chain is 8m (25ft); ability to swivel so chain does not shorten; padding on chain to avoid rubbing; grass to eat; moving once a day; habituated to tethering and collar; water restricted (brought in buckets every 6 hours); access to grazing. | |

| | | |
|---|---|---|
| | **DOMAIN** | |
| | **1** | *Impact assessed separately under diet.* |
| | **2** | Minimal tactile contact with neighbour. |
| | **3** | Lack of shelter may be associated with heat stress and sunburn and possibly with increased eye problems.<br>Risk of poisoning. |
| | **4** | Must learn to drink when water is made available.<br>Lack of ability to escape potential predators. |
| **16** | Outdoor tethering: – Partial social contact, no shelter.<br>**Assumptions:** Visual but not tactile contact.<br>**DOMAIN** | |
| | **1** | *Impact assessed separately under diet.* |
| | **2** | Less contact than “Outdoor tethering – No social contact, no shelter”. |
| | **3** | As for “Outdoor tethering – No social contact, no shelter”. |
| | **4** | As for “Outdoor tethering – No social contact, no shelter”. |

**Table S4.** Assumptions and notes made when panellists considered interventions related to foundation training.

| **Duration** | Up to one1 hour (per day). |
|---|---|

Pre-workshop assumptions: Assume positive reinforcement where relevant; interventions are gradually introduced. For all interventions that are repeated on a daily/weekly basis (e.g., training and work), assessment is of one day’s training or work. Bit introduction is assumed to be manual (in-hand), rather than the bit being attached to side reins and the horse left unattended.

| | **Assumptions** |
|---|---|
| a | Positive reinforcement (scratching, petting, rubbing). |
| b | Interventions are gradually introduced. |
| c | Skilled handler (timing of release of pressure). |
| d | Familiar environment. |
| e | Safe enclosure and underfoot substrate. |
| f | Appropriate well-fitted gear and soft ropes. |
| g | Familiar handler. |
| h | Habituated to basic handling. |
| i | Horse healthy and has had teeth checked. |

| | **Notes** | Sandy yard/arena, type of ropes: soft or leather, horse has been trained to lead but not habituated to being tied up. |
|---|---|---|
| **1** | Dropping horse with ropes (using traction to bring a horse to the ground).<br>**DOMAIN** | |
| | **1** | – |
| | **2** | Retrain, unpredictable event, dust, body posture, rope on skin. |
| | **3** | Injuries: leg, neck, back, joints, bruising, rope burns, muscle tension/tears, irritants in eyes, nose, respiration, GI depending on duration. |

| | | |
|---|---|---|
| | **4** | Isolation from other horses. Possibility of learned helplessness. |
| **2** | Advance and retreat.<br>**DOMAIN** | |
| | **1** | – |
| | **2** | – |
| | **3** | – |
| | **4** | Constraint on horse-horse interactions, limited avoidance responses. |
| **3** | Round pen training.<br>**Assumptions:** Chasing horses, using ropes, whips or flags, with or without physical contact being made.<br>**DOMAIN** | |
| | **1** | – |
| | **2** | Unfamiliar environment with possibility of dust hazard. |
| | **3** | Leg injuries, falling, respiration of dust, rhabdomyolysis. |
| | **4** | Isolation from other horses. Some confinement. |
| **4** | Pressure and release training.<br>**DOMAIN** | |
| | **1** | – |
| | **2** | – |
| | **3** | – |
| | **4** | Isolation from other horses unless multiple horses are being trained at once. |
| **5** | Bit introduction.<br>**Assumptions:** In-hand with trainer present and observing, rather than unobserved.<br>**DOMAIN** | |
| | **1** | – |
| | **2** | – |
| | **3** | – |
| | **4** | Tongue movement, swallowing and chewing may be affected. |
| **6** | Bit habituation.<br>**Assumptions:** Reins attached but not fixed, no noseband.<br>**DOMAIN** | |
| | **1** | Food withheld. |
| | **2** | – |
| | **3** | Risk of some pain to mouth and injuries from bit. |
| | **4** | Restriction to movement, tongue movement, swallowing, chewing.<br>Loss of choice about where to go within enclosure. |
| **7** | Saddle and girth habituation.<br>**DOMAIN** | |
| | **1** | – |
| | **2** | Familiar environment, unpredictable event. |
| | **3** | Injury, falling over, stress may lead to gastric ulceration. |
| | **4** | Saddle and girth pressures may mimic predatory stimuli. |

| | | |
|---|---|---|
| **8** | a. Backing <18 months.<br>**Assumptions:** Horse has been long reined or driven.<br>**DOMAIN** | |
| | **1** | – |
| | **2** | – |
| | **3** | Physical development may not be ready to carry a rider. |
| | **4** | Saddle and girth pressures may mimic predatory stimuli. |
| **9** | b. Backing >18 months.<br>**Assumptions:** Horse has been long reined or driven.<br>**DOMAIN** | |
| | **1** | – |
| | **2** | – |
| | **3** | Physical development may be better able to carry a rider. |
| | **4** | Pressures may mimic predatory stimuli. |
| **10** | Forced flexion (using force to flex the horse’s neck).<br>**Assumptions:** Side-reins attached to girth or surcingle, bit and reins (elastic), forced flexion, at the vertical.<br>**DOMAIN** | |
| | **1** | – |
| | **2** | – |
| | **3** | Neck, muscle, swallow, eat or drink, injuries, tail injuries, when tied to tail, falling. |
| | **4** | Restrict movement and isolation from other horses. |
| **11** | Hobbling.<br>**Assumptions:** Forelimbs hobbled together, padded or soft leather hobbles, hobbles are the width of normal distance between left and right cannon bones.<br>**DOMAIN** | |
| | **1** | – |
| | **2** | – |
| | **3** | Injuries, falling over, running into objects, rope burns, muscle pull, leg injuries. |
| | **4** | Significant restriction of movement and isolation from other horses. |

**Table S5.** Assumptions and notes made when panellists considered interventions related to medical interventions.

| | |
|---|---|
| **Duration** | Hours, one-off procedure. |

| | **Assumptions** |
|---|---|
| a | Best practice, surgical intervention will be discussed in another section, familiar location. |
| b | Horse otherwise healthy, adult, used to handling, used for ridden work, pain is manageable during examination. |
| c | Best practice preventative health care in place. |
| d | Owner compliant and financially capable. |

| | |
|---|---|
| e | Assume first visit. |
| f | No other professional intervention. |
| g | Sedation only after primary diagnostic interventions, food would be withheld after sedation. |

| | **Notes** | |
|---|---|---|
| **1** | Medical – One-off, immediately curative. Considered spasmodic colic as primary example.<br>**DOMAIN** | |
| | **1** | Test willingness to eat, withhold food (exception for donkeys). |
| | **2** | No access to edible bedding. |
| | **3** | Negative impact from veterinary intervention, rectal examination/temperature, some unfamiliar auscultation/palpation of sensitive areas, examination of gums and tongue, nasal gastric tube and paracentesis. |
| | **4** | Fear, stress, pain from the interventions and physical restraint. |
| **2** | Medical – Repeated short-term curative. Considered fresh superficial mid-cannon injury to a forelimb as primary example.<br>**DOMAIN** | |
| | **1** | – |
| | **2** | Confinement – see consideration of individual stable with no social contact and no yard access. |
| | **3** | TMPS, examination and treatment inducing pain. |
| | **4** | Confinement – see consideration of individual stable with no social contact and no yard access. |
| **3** | Medical – Repeated long-term curative. Considered laminitis as primary example, also discussed equine gastric ulcer syndrome, equine herpes virus, equine influenza, colitis, diarrhoea, hyperlipaemia, minor tendon injuries and weight loss.<br>**DOMAIN** | |
| | **1** | Food withheld. |
| | **2** | Confinement – see consideration of individual stable with no social contact and yard access |
| | **3** | Inducing pain – foot lifting, hoof-testers, foot supports, application of therapeutic supports, manipulations for radiography, venepuncture. |
| | **4** | Restraint, low-grade fear to novel stimulus. Frustrated foraging behaviours. Confinement – see consideration of individual stable with no social contact and yard access. |
| **4** | Medical – Prolonged palliative treatment. Considered Sweet Itch/Queensland Itch as primary example, also discussed chronic obstructive pulmonary disease<br>**DOMAIN** | |
| | **1** | – |
| | **2** | Confinement during dusk and evening in midge season. See consideration of individual Stable with no social contact and no yard access. |
| | **3** | Venepuncture, skin scrapings/biopsy, intradermal allergy testing. |
| | **4** | Protective rugs may inhibit some normal behaviours. |

**Table S6.** Assumptions and notes made when the panellists considered interventions related to surgical interventions.

| | |
|---|---|
| **Duration** | Hours, one-off procedure. Pre-anaesthetic, Induction, Surgery, Recovery, Post-operative Period (until wound healed 7 days). |

| | **Assumptions** |
|---|---|
| a | Best practice (Veterinarian is up-to-date with continuing professional development and who has experience with this surgery). No financial constraints for owner. Diagnosis is confirmed. |
| b | Preoperative analgesia 1 hour before surgery, post-operative analgesia for 5 days (except for hoof abscess). |
| c | Diagnosis established (e.g., not considering impact of hoof-testers in locating hoof abscess). |
| d | Infection control following British Equine Veterinary Association guidelines on misuse of antibiotics. |
| e | Owner compliant with post-operative care. Stable and stable management are at an industry standard level, non-enriched environment (home stable or vet hospital), clean straw or shavings for bedding, good-quality air circulation, other horses visible, but no contact. |
| f | Adult horse in working condition, and otherwise healthy, including vaccinations etc. Planned surgery, except in the case of colic. |
| g | General Anaesthetic (GA) induction in padded knockdown room, surgery in adjacent theatre, move into theatre by hobble hoist, padded operating table, no environmental management of theatre (no air filtration, etc., but clean room), gaseous anaesthesia (isoflurane), endotracheal tube. Recovery is the reverse of induction (off table by hoist into knockdown box etc.) |

| | **Notes** | |
|---|---|---|
| **1** | Surgical single curative minor. Consideration revolved around Hoof Abscess (Pus in foot) as an example. Non weight bearing on one leg– Hoof abscess– Paring, resection near sensitive tissues (potential for pain but no entry into sensitive tissues), pus is drained, poultice dressing, intravenous non-steroidal anti-inflammatory drugs (NSAID) at time of treatment, oral NSAID pain relief for approximately 4 more days. Wet winter so animal confined in stable after procedure for duration (7 days) of intervention being considered.<br>**DOMAIN** | |
| | **1** | No withholding of food or water. |
| | **2** | Confinement in familiar stall/stable for 7 days. |
| | **3** | Pain from lancing. |
| | **4** | Impact of confinement – refer to Housing. |
| **2** | Surgical single curative intra-cavity surgery or repeated minor surgery. Consideration revolved around Inguinal Cryptorchid surgery as an example.<br>General Anaesthetic (GA). Protocol as per assumptions above, para-inguinal incision, Dorsal recumbency.<br>Withholding of food (6 hours) pre-op.<br>Withholding of water (1 hour) pre-op.<br>Post-operative return to normal feeding after recovery from GA effects (3 hours post standing).<br>Discharge assumed at 7 days, best practice might be sooner.<br>**DOMAIN** | |
| | **1** | Hunger. |
| | **2** | Confined for induction and recovery in unfamiliar environment.<br>Unfamiliar odours. |

| | | |
|---|---|---|
| | | Surface is unstable.<br>Hospital environment unfamiliar. |
| | **3** | Residual pain.<br>Risk of injury for recovery.<br>Paralytic ileus.<br>Dysphoria (GA-induced effects).<br>Risk of complications. |
| | **4** | Frustration.<br>Poor balance. |
| **4** | Surgical major deep intra-cavity surgery– Consideration revolved around Colic Small intestine torsion with resection and anastomosis as an example. No pre-operative starvation, otherwise GA procedure as per assumptions. No exercise or grazing post-operative though some practices do introduce grazing and hand-walking earlier. Assume hospitalisation provides more intensive care than standard hospital stable therefore the environment and non-contact exposure to other animals might be less than other hospital stables. Intensive care stable has drip lines, lights and heaters.<br>**DOMAIN** | |
| | **1** | Withholding of food and water (24 hours post-operative). More hunger than other surgical interventions.<br>IV fluids to balance hydration. |
| | **2** | As for other forms of confinement in hospital but with greater restriction of movement due to attachment to drip lines and more frequent interventions for nursing. |
| | **3** | Indwelling catheter.<br>Stomach tubes for testing reflux three times daily (this may taper off after the first few days once ileus resolves).<br>Risk of ill-health through toxicity of drugs.<br>Risk of laminitis.<br>Risk of hyperlipaemia (e.g., in Highland ponies).<br>Risk of dying (surgery might not be curative).<br>Risk of pain, visceral and from wound itself. |
| | **4** | More interaction with unfamiliar people.<br>Less ability to lie down to rest and sleep. |

**Table S7.** Assumptions and notes made when the panellists considered interventions related to elective procedures.

| | |
|---|---|
| **Duration** | Castration – up to 1 hour, one-off procedure.<br>Identification minutes, one-off procedure except hoof –branding.<br>Other elective procedures minutes, one-off procedure. |

Pre-workshop assumptions: Sedation and local anaesthetic; no flies (winter); opportunity to move around (to reduce swelling); carried out <1 year of age. For castration without veterinary supervision without post-op analgesia assume no controlled drugs are used.

| | **Assumptions:** (Castration). |
|---|---|
| a | Sedation, analgesia and local anaesthetic (excluding "Castration without veterinary supervision without post-op analgesia" and wolf tooth removal). |
| b | Best practice as described on *Equine Clinical Medicine: Surgery and Reproduction* p.375 |
| c | No flies (winter). |
| d | Opportunity to move around to reduce swelling. |

| | |
|---|---|
| e | Carried out at <1 year of age, previously with conspecific companions. |
| f | Monitoring anaesthetic dose to effect. |
| g | Familiar location (except Hobday's and Modified Forsell's procedures, which are expected to take place in hospital) that is a clean, open space or padded enclosure. |
| h | Aseptic technique. |
| i | Horse is vaccinated against tetanus. |
| j | Surgeon familiar with specific procedure. |
| k | Horse is otherwise healthy, well-cared-for and compliant. |
| l | Post-operative treatment and monitoring: 6-12hours in clean box. Post-operative analgesia for 5 days. |
| m | Assessments are for single intervention (i.e., first time, no repeats). |

| | **Assumptions:** (Identification) |
|---|---|
| a | Best practice. |

| | **Assumptions:** (Other elective procedures) |
|---|---|
| a | Best practice. |
| b | Carried out at <1 year of age.<br>Duration of assessment for 7 days post-op. |

| | **Notes** *(record any variations to duration here)* | |
|---|---|---|
| **1** | Standing castration with post-op analgesia.<br>**Assumptions:**<br>*Not considering impact of loss of testicles.*<br>**DOMAIN** | |
| | **1** | – |
| | **2** | – |
| | **3** | Risks of infection, eventration and haemorrhage (1-in-10 incidence across all complications).<br>Pain during and after procedure.<br>Inflammation. |
| | **4** | Restraint.<br>Delay before return to normal behaviour.<br>Inhibition of some behaviours due to pain. |
| **2** | Standing castration without post-op analgesia.<br>**DOMAIN** | |
| | **1** | – |
| | **2** | – |
| | **3** | As for "Standing castration with post-op analgesia". |
| | **4** | As for "Standing castration with post-op analgesia" but expect increased latency to return to normal behaviour. |
| **3** | Castration with general anaesthetic and post-op analgesia.<br>**Assumption:** Performed within home environment. No sutures. 20m GA. | |

| | | |
|---|---|---|
| | (Assessed relative to "Standing castration with post-op analgesia")<br>**DOMAIN** | |
| | **1** | _ |
| | **2** | _ |
| | **3** | As for "Standing castration with post-op analgesia" but increased risk of injury during induction (with the horse being ataxic when going down).<br>Myopathy during GA.<br>Increased risk injury in post-GA recovery.<br>Slightly increased risk of contamination leading to local infection.<br>Risk of intestinal stasis. |
| | **4** | Latency to return to normal behaviour in 24 hours or longer.<br>Risk of sedative wearing off before ketamine. |
| **4** | Castration with general anaesthetic but without post-op analgesia.<br>**DOMAIN** | |
| | **1** | – |
| | **2** | – |
| | **3** | As for "Castration with general anaesthetic with post-op analgesia". |
| | **4** | As for "Castration with general anaesthetic with post-op analgesia". |
| **5** | Castration without veterinary supervision, without post-op analgesia (no controlled drugs used).<br>**Assumptions:** In bush (= home environment). Horse immobilised by physical restraint (i.e., best-practice roping).<br>**DOMAIN** | |
| | **1** | – |
| | **2** | – |
| | **3** | Crushing of tissues.<br>Neurological wind-up.<br>Increased risk of injury during restraint and escape attempts. |
| | **4** | Severe hyperalgesia. |
| **6** | Hot branding.<br>**Assumptions:** Two locations at least three possible for symbols. Location assessed=off-side shoulder (*see Horse Sense*).<br>Sensitivity may be slightly lower on the rump than on the shoulder.<br>After weaning at 5-8 months of age.<br>No pain relief.<br>**DOMAIN** | |
| | **1** | – |
| | **2** | – |
| | **3** | Local inflammation, still some after 24 hrs. Inflammation at 7days.<br>Scarring. |
| | **4** | Behavioural reactions consistent with pain and distress during the operation.<br>Local hyperalgesia for 24hrs.<br>Increased dermal sensitivity away from branding site. |
| **7** | Freeze branding.<br>**DOMAIN** | |
| | **1** | – |

| | | |
|---|---|---|
| | **2** | – |
| | **3** | Local inflammation, still some after 24hrs. Inflammation at 7days.<br>Scarring. |
| | **4** | Less behavioural reaction consistent with pain and distress than for hot branding. |
| **8** | Hoof branding.<br>**Assumptions:** Not near sensitive tissue. More than 4 numbers applied in the brand. Duration of procedure = Few seconds.<br>(NB Must be repeated).<br>**DOMAIN** | |
| | **1** | – |
| | **2** | – |
| | **3** | Secondary risk that "hoof can chip away". |
| | **4** | Minimal behavioural reactions. |
| **9** | Microchipping.<br>**Assumptions:** Clipping of hair-coat; superficial and deep local analgesia.<br>Chip introduced into nuchal ligament via a 12-gauge needle.<br>Performed at approximately 6 months of age.<br>**DOMAIN** | |
| | **1** | – |
| | **2** | – |
| | **3** | Increased skin temperature at microchip site and more general (Erber et al. ref 2011 c/o LN).<br>Low-risk foreign body reaction: bruising and abscess formation.<br>Low-risk migration of microchip.<br>(C.P. reported cases of soft-tissue sarcomas in cats/dogs). |
| | **4** | Behavioural reaction to chip insertion.<br>LA tolerated reasonably well (NdB pers. obs).<br>No evidence identified by group of behavioural effect after 24 hours unless complications. |
| **12** | Caslick's procedure.<br>**Assumptions:** The procedure creates an elective wound that repairs by primary intention.<br>Fine strip of vulval skin above pelvic shelf is removed and the exposed tissues are sutured together.<br>(NB at some point sutured tissues will need to be opened up again if bred – i.e. a further incision).<br>(If bred, Caslick's is likely to be repeated, with breeding cycle, for rest of breeding life).<br>**DOMAIN** | |
| | **1** | – |
| | **2** | – |
| | **3** | – |
| | **4** | Some difficulty urinating – increased frequency, low volume urination/intermittent stream for up to 4 days. |
| **13** | Modified Forsell's procedure (= removal of 2cm of motor nerves rather than muscles, as in the original Forsell's procedure).<br>**Assumptions:** Crib-biting horse.<br>Operation may be 10 minutes in duration for both sides of the neck.<br>NB No longer recommended (i.e., difficult to define best practice).<br>**DOMAIN** | |

| | | |
|---|---|---|
| | **1** | Decreased food intake if impact on ingestive behaviour and possible on the consummatory aspect of crib-biting itself. |
| | **2** | – |
| | **3** | Low risk of neuroma. |
| | **4** | Preventing a motivated behaviour (at least for 7days).<br>Most recent studies suggest that no impact on performance of crib-biting.<br>Interference with feeding behaviour. |
| **14** | Hobday's procedure.<br>**Assumptions:** Standing, restrained, sedated, tubed, laser, peri-operative local anaesthesia.<br>**DOMAIN** | |
| | **1** | Post-operative mild/moderate reduction in food intake. |
| | **2** | – |
| | **3** | Wound and some post-operative inflammation. |
| | **4** | Some difficulty in swallowing. |
| **15** | Wolf teeth removal.<br>**Assumptions:** No analgesia. Assessment based on the size of tooth expected in a 1-year-old.<br>**DOMAIN** | |
| | **1** | Some reduction in food intake. |
| | **2** | – |
| | **3** | Risk of tooth root retention.<br>Risk of fracture.<br>Risk of post-op infection. |
| | **4** | Possible evidence of some post-op depression. |
| **16** | Clitorectomy.<br>**Assumptions:** No sutures.<br>NB Limited justification, especially regarding Contagious Equine Metritis.<br>**DOMAIN** | |
| | **1** | – |
| | **2** | – |
| | **3** | Risk of damage to sensitive well-innervated area.<br>Risk of some haemorrhage.<br>Risk of infection, swelling, urethral damage – higher than Caslick's. |
| | **4** | Some difficulty in urinating. |

**Table S8.** Assumptions and notes made when panellists considered interventions related to care procedures.

| **Duration** | Minutes to hours. |
|---|---|

Pre-workshop assumptions: Horse is compliant.

| | **Assumptions** |
|---|---|
| a | Assume horse is approachable and compliant with normal handling. |
| b | Procedure is carried out following appropriate desensitisation and/or habituation (may not apply to all interventions – note exceptions). |

| | |
|---|---|
| c | Adult horse that has been used for riding. |
| d | Assume familiar environment. |
| e | Horse is well hydrated and well fed prior to restraint and commencement of procedure. |
| f | Procedure is carried out by competent technician. |

| | Notes | |
|---|---|---|
| **1** | Whisker removal.<br>**Assumptions:** Whiskers are cut (not pulled out) from around muzzle only.<br>**Note:** Banned in some countries to remove hairs with protective/sensory function.<br>**DOMAIN** | |
| | **1** | – |
| | **2** | – |
| | **3** | – |
| | **4** | Possible some proprioceptive deficits and reduced spatial awareness during grazing due to removal of sensory hairs. |
| **2** | Clipping.<br>**Assumptions:** Full body clip (including legs, head but NOT whiskers); rugged.<br>**DOMAIN** | |
| | **1** | – |
| | **2** | Challenge to thermal regulation.<br>Noise of clippers during procedure. |
| | **3** | Potential increased risk of injury during clipping.<br>Potential increased risk of injury post-clipping.<br>Risk of burns, cuts from clipping procedure. |
| | **4** | Horse is more sensitive to tactile stimulation.<br>Lack of ability to self-regulate thermal comfort/rugging. |
| **10** | Dental – Rasping/floating using manual tools.<br>**Assumptions:** Hausmann's gag used.<br>**DOMAIN** | |
| | **1** | – |
| | **2** | – |
| | **3** | Risk of traumatic injury to soft tissue and joints. |
| | **4** | Aversive procedure – potential for horse to become head/mouth shy.<br>Cannot close mouth and swallow effectively during procedure. |
| **14** | Horse-walking machines.<br>**Assumptions:** Mechanical rotary; horses are not tied; no electric shocks (aligning with ISES principles and position statement on the use of aversive stimuli); attendant present observing horses; suitable footing surface; with other familiar horses; walking pace.<br>**Duration** 30 minutes (15 in each direction).<br>**DOMAIN** | |
| | **1** | – |
| | **2** | Restricted barren environment in contrast to being walked on a lead rope. |
| | **3** | Risk of injury from equipment. |
| | **4** | Inability to regulate pace, perform other locomotory behaviours or stop.<br>Limited capacity for flight response. |

| | | |
|---|---|---|
| | | No physical interaction with other horses. |
| **15** | Rugging (in winter).<br>**Assumptions:** Unclipped paddock horse; checked and removed once/day; correct sized rug.<br>**DOMAIN** | |
| | **1** | – |
| | **2** | Challenge to thermal regulation.<br>Challenge to physical comfort. |
| | **3** | Increased risk of abrasion and some types of skin infection/infestation (lice).<br>Increased risk of injury (e.g., when straps/rugs become caught on fencing). |
| | **4** | Reduced capacity for allogrooming.<br>Lack of ability to self-regulate thermal comfort. |
| **16** | Hoods.<br>**Assumptions:** Standard practice as per (*see* p.29 *Horse Sense*).<br>Observer makes regular checks.<br>**DOMAIN** | |
| | **1** | – |
| | **2** | Challenge to thermal regulation.<br>Challenge to physical comfort. |
| | **3** | Small risk of injury during placement of hood if horse panics.<br>Increased risk of abrasion and some types of skin infection/infestation (lice).<br>Increased risk of injury (e.g., when hood becomes caught on fencing). |
| | **4** | Risk of altered response from other horses due to changed appearance.<br>Reduced ability for allogrooming, self-grooming and possibly fly-swatting.<br>Lack of ability to self-regulate thermal comfort. |
| **17** | Grazing muzzles.<br>**Assumptions:** Fits correctly; quick release if caught; horse can eat limited quantities of forage (trickle grazing); drink as normal.<br>**Duration** 24/7 for 5 months<br>**DOMAIN** | |
| | **1** | Reduces food intake. |
| | **2** | – |
| | **3** | Small rub sores. |
| | **4** | Restriction on grazing and browsing including limits on prehension.<br>Restriction on allogrooming and self-grooming.<br>Some horses rub or paw at muzzle.<br>Possible frustration and reduced oral satisfaction from the limit on grazing. |
| **18** | Collars for oral windsuckers/crib-biters.<br>**Assumptions:** Design has rigid structure to accommodate the trachea; no electric shock.<br>**DOMAIN** | |
| | **1** | – |
| | **2** | – |
| | **3** | Risk of pain during swallowing.<br>Risk of airway obstruction.<br>Risk of cardiovascular obstruction (fainting).<br>Risk of injury if caught or snagged.<br>Risk of rubbing and abrasions.<br>Risk of swelling. |

| | | |
|---|---|---|
| | **4** | Risk of reduced swallowing behaviour.<br>Frustration from reduction in capacity to crib-bite without addressing cause. |
| **19** | Restrictive (cranked) nosebands.<br>**Assumptions:** No space under noseband; single bit; worn during ridden work; crank Cavesson noseband with padding.<br>**Duration** one hour of work.<br>**DOMAIN** | |
| | **1** | – |
| | **2** | – |
| | **3** | Increased risk of ulceration in the mouth.<br>Potential for reduced vascular perfusion of tissues distal to noseband.<br>Potential for micro-fractures of nasal bone.<br>Potential for skin lacerations around jaw. |
| | **4** | Compromised ingestion behaviour.<br>Inability to open jaw.<br>Chewing and yawning not possible.<br>Possible restriction of swallowing. |
| **20** | Tongue-ties.<br>**Assumptions:** Tie is used during racing (flat, jumps, harness) track-work and race days; tightened to prevent tongue moving.<br>**Duration** up to one hour.<br>**DOMAIN** | |
| | **1** | – |
| | **2** | – |
| | **3** | Dry tongue and dry oral anatomy distal to the tongue tie.<br>Tongue trauma/lacerations.<br>Potential for reduced vascular perfusion of localised tissues.<br>Potential for damage to hyoid bones. |
| | **4** | Reduced capacity to swallow.<br>Cannot eat or drink. |
| **22** | Deworming.<br>**Assumptions:** Paste, single dose; mildly unpalatable.<br>**DOMAIN** | |
| | **1** | – |
| | **2** | – |
| | **3** | Risk of some tissue damage through killing worms. |
| | **4** | Potential for head shyness.<br>Behaviours indicative of unpalatability. |
| **23** | Pulling the mane and tail (manual depilation for cosmetic purposes).<br>**Assumptions:** Pulling whole mane and tail at one time; horse is warm; week before competition; hay net provided (*see* p.34 *Horse Sense*).<br>**Duration** 2 hours.<br>**DOMAIN** | |
| | **1** | – |
| | **2** | – |
| | **3** | Localised trauma. |

| | | |
|---|---|---|
| | **4** | Localised pain.<br>Large individual variation in behavioural responses.<br>Less effective swishing to remove flies after procedure. |
| **39** | Sheath cleaning.<br>**Assumptions:** Conducted on gelding; no pre-existing infection/inflammation.<br>Using proprietary sheath-cleaning product according to label instructions.<br>**DOMAIN** | |
| | **1** | – |
| | **2** | – |
| | **3** | Risk of introducing infections.<br>Allow infections to establish due to damage to microflora.<br>Risk of trauma from removal of inspissated smegma from urethral fossa. |
| | **4** | – |
| **41** | Trimming.<br>**Assumptions: (***See* p.21 *Horse Sense*); trimming procedure and regularity as for shoeing; permanently barefoot horse.<br>**Note:** There are many different approaches to trimming.<br>**DOMAIN** | |
| | **1** | – |
| | **2** | – |
| | **3** | Mild changes to angulation and length of hoof.<br>Sole close to ground after trimming; exposure to potential bruising. |
| | **4** | Movement restricted by leg lifting. |
| **44** | Shoeing – cold.<br>**Assumptions:** Regular (6-week) shoeing; off-the-shelf shoes that fit; at home; good footing surface; toe clips.<br>**Duration** about 1 hour for all 4 feet.<br>**DOMAIN** | |
| | **1** | – |
| | **2** | – |
| | **3** | Movement restricted by leg lifting.<br>Minor risk of penetration injury and secondary infection from nail.<br>Potential for weakening of the hood wall.<br>Very minor risk of lacerations to opposing limb.<br>Potential sensitivity when horse has minor lameness.<br>Adjustment to foot position with change of shoes.<br>Some reduction in foot flexibility in contrast to barefoot.<br>Possible reduction in correct fitting compared with hot shoeing. |
| | **4** | Some horses may be "foot sore".<br>Loss of frog contact may reduce proprioception. |
| **45** | Shoeing – hot.<br>**Assumptions:** Regular (6-week) shoeing; shoes that fit; at home; good footing surface; toe clips.<br>**Duration** about 1 hour for all 4 feet.<br>**DOMAIN** | |
| | **1** | – |
| | **2** | Transient smoke, noise and odour. |

| | | |
|---|---|---|
| | **3** | Movement restricted by leg lifting.<br>Minor risk of accidental burn or thermal injury.<br>Minor risk of penetrating injury and secondary infection from nail.<br>Potential for weakening of the hoof wall.<br>Very minor risk of lacerations to opposing limb.<br>Potential sensitivity when horse has minor lameness.<br>Adjustment to foot position with change of shoes.<br>Some reduction in foot flexibility in contrast to barefoot. |
| | **4** | Some horses may be “foot sore”.<br>Loss of frog contact. |

**Table S9.** Assumptions and notes made when the panellists considered interventions related to restraint procedures.

| | |
|---|---|
| **Duration** | Specified under each intervention. |

Pre-workshop assumptions: Horse is compliant.

| | **Assumptions** |
|---|---|
| a | Assume horse is approachable and compliant with normal handling. |
| b | The impact is scored based on the first time intervention applied to a given horse. |
| c | Adult horse that has been used for riding. |
| d | The environment is familiar. |
| e | Horse is well hydrated and fed prior to restraint. |

| | | |
|---|---|---|
| | **Notes** | |
| **1** | Ear twitch.<br>**Duration** best practice maximum of 15 minutes (prior to arousal).<br>**DOMAIN** | |
| | **1** | – |
| | **2** | – |
| | **3** | Potential for pain and damage.<br>Massive damage and pain in donkeys (including cartilage damage).<br>Potential for injury when behaviour is violent. |
| | **4** | All forms of restraint restrict movement/placing a high cost on movement.<br>Potential for ear -shy behaviour (short and long term).<br>Threshold for compliance may be reached (acquiescence turns to violent behaviour). |
| **2** | Nose twitch.<br>**Duration** best practice maximum of 15 minutes (prior to arousal).<br>**DOMAIN** | |
| | **1** | – |
| | **2** | – |
| | **3** | Initial pain may lead to increase in endogenous endorphins. |
| | **4** | Provides physical control as well as behavioural restriction. |
| **3** | Skin twitch. | |

<table>
<tr><td rowspan="5"></td><td colspan="2">Duration maximum 5 minutes (prior to arousal).<br>DOMAIN</td></tr>
<tr><td>1</td><td>–</td></tr>
<tr><td>2</td><td>–</td></tr>
<tr><td>3</td><td>Less physical damage than ear/nose twitches.</td></tr>
<tr><td>4</td><td>Note: Practitioners state that it has a distracting effect thus allowing other minor painful procedures to be conducted.<br>Potential “overshadowing”.<br>Unlikely to be pain-free.</td></tr>
<tr><td rowspan="5">4</td><td colspan="2">Chifney bit, also known as an “inverted port” or ‘stallion bit”.<br>Best standard practice is to just vibrate and release, rather than pull/tug; but the absolute absence of any pulling or tugging seems unlikely.<br>Duration 15 minutes maximum.<br>DOMAIN</td></tr>
<tr><td>1</td><td>–</td></tr>
<tr><td>2</td><td>–</td></tr>
<tr><td>3</td><td>Risk of damage to mouth if horse reacts or unpredictable event occurs.<br>Note: high risk of damage to mouth if used inappropriately.</td></tr>
<tr><td>4</td><td>–</td></tr>
<tr><td rowspan="5">6</td><td colspan="2">Tongue holding/pulling.<br>Duration one minute maximum.<br>DOMAIN</td></tr>
<tr><td>1</td><td>–</td></tr>
<tr><td>2</td><td>–</td></tr>
<tr><td>3</td><td>Potential for damage to tongue.<br>Potential fracture of the hyoid bones.</td></tr>
<tr><td>4</td><td>Risk of head shyness?<br>Restriction on swallowing behaviour.</td></tr>
<tr><td rowspan="5">7</td><td colspan="2">Leg lifting.<br>Assumptions: Manual or quick-release strap; front leg.<br>Duration one minute maximum.<br>DOMAIN</td></tr>
<tr><td>1</td><td>–</td></tr>
<tr><td>2</td><td>–</td></tr>
<tr><td>3</td><td>Low risk of injury where lift can be released immediately.<br>Lift needs to be at comfortable height for the animal (not human) or animal will lose balance.</td></tr>
<tr><td>4</td><td>–</td></tr>
<tr><td rowspan="3">8</td><td colspan="2">Stabilizer™ (chain under upper lip). Chain applying pressure onto gum of maxilla under the upper lip. Best practice is to apply sustained low pressure, but mostly used punitively with excessive pressure; some designs will have tubing on the chain.<br>Duration 15 minutes maximum.<br>DOMAIN</td></tr>
<tr><td>1</td><td>–</td></tr>
<tr><td>2</td><td>–</td></tr>
</table>

| | | |
|---|---|---|
| | **3** | Damage from the chain likely when used with excessive pressure. |
| | **4** | – |
| **9** | Slings (for veterinary purposes).<br>**Assumptions:** Specially designed for horses; food and water available.<br>**Duration** 7 days maximum (post-surgery recovery).<br>**DOMAIN** | |
| | **1** | Diet needs to be modified. |
| | **2** | Novel environment. |
| | **3** | Impact affected by use post-operatively (overshadowed by other recovery factors).<br>Muscle wastage.<br>Risk of faecal soiling.<br>Risk of pressure sores. |
| | **4** | Limited social contact (sling should be located where there are other horses).<br>**Note**: Sling allows horse to be upright compared to no sling (positive benefit) but movement is necessarily restricted. |
| **10** | External restraint: Use of crush, spa, padded boxes.<br>**Assumptions:** Limited social contact (other horses around in sight); fixed size/walls (*see* p.281 *Horse Sense*); non-slip flooring.<br>**Duration** 1 hour maximum.<br>**DOMAIN** | |
| | **1** | No access to food and water. |
| | **2** | Movement restricted (cannot turn around). |
| | **3** | Low risk of injury. |
| | **4** | Behaviour limited (due to restraint). |

**Table S10.** Assumptions and notes made when panellists considered interventions related to road transport.

| | |
|---|---|
| **Duration** | Up to 6 hours |

Pre-workshop assumptions: Vehicle designed to transport horses; Food and water rest stops are provided every 6 hours; Head height is not fixed; Horses are habituated to loading and transport; Journey time does not exceed 6 hours.

| | **Assumptions** |
|---|---|
| a | Vehicle designed to transport horses (non-slip flooring).<br>**Note:** Design of horse-transport boxes are not necessarily appropriate for horses. No standard available. Massive differences between transport purpose (e.g., transport for slaughter and transport for recreation). |
| b | Head height is not fixed. |
| c | Horses are have learned to tolerate loading and transport.<br>**Note:** much transport of grouped horses is of horses not habituated to transport. |
| d | Journey time does not exceed 6 hours. |
| e | Driver is competent (regarded as unusual). |
| f | No access to water. |
| g | Sawdust provided (as flooring to soak-up urine rather than as bedding). |

| | Notes | |
|---|---|---|
| **1** | Individual.<br>**Assumptions:** Trailer/horse float pulled behind a vehicle; protection boots and tail wrap; hay net provided.<br>**DOMAIN** | |
| | **1** | Restriction on water intake. |
| | **2** | Lack of control over thermal environment (discomfort).<br>Ventilation compromised when back doors are closed.<br>Opening doors may increase dust levels.<br>Forced ventilation is best practice but not standard for this type of transporter. |
| | **3** | Risk of injury from road traffic accident (outside of driver's control).<br>Risk of injury where ramps have gaps between floor of trailer/ramp or ramp/ground.<br>Restricted movement has positive benefits in terms of safety.<br>Risk of strangulation when horses are tied; length of rope is crucial but guidance is weak in terms of what is appropriate.<br>Where horses are standing through journey there may be a risk of exhaustion due to requirement for balancing throughout journey.<br>Risk of thermal stress (hypothermia and hyperthermia).<br>Urinary retention due to reluctance to urinate or inability to assume appropriate posture during transit. |
| | **4** | Restricted movement; adoption of fixed posture.<br>Challenge from lights when driving at night, volume and aversive noise from other vehicles.<br>Even habituated horses can have some behavioural problems with loading and unloading.<br>Some horses will have learned that transport is unpleasant.<br>Loading process may be associated with learned fear of human loaders and goading methods during loading.<br>Lack of social interactions as a result of partial or complete isolation.<br>Colic risk increases in unseasoned travellers then decreases with journey frequency. |
| **2** | Group – with familiar companions – individually penned.<br>**Assumptions:** Horses in group are loaded together and compatible; lorry/truck; each horse has access to hay net.<br>**DOMAIN** | |
| | **1** | Restriction on water intake. |
| | **2** | Generally tied-up with short leads to reduce potential for food competition.<br>Increased ammonia concentrations relative to individual transportation. |
| | **3** | Increased infectious disease risk due to stress and social contact. |
| | **4** | Partial social contact (visual and olfactory); no tactile contact (usual practice inhibits contact because of grilles between horses).<br>May be general stress reduction due to presence of familiar conspecifics.<br>Increase in thermal stress from shared body heat.<br>Potential for indirect aggressive behaviour from neighbours (food competition).<br>Increased aggression because of close confinement. |
| **3** | Group – with unfamiliar companions – individually penned.<br>**DOMAIN** | |
| | **1** | Restriction on water intake. |
| | **2** | Higher ammonia concentrations due to increased stress from unfamiliar animals. |

| | | |
|---|---|---|
| | **3** | High risk of infectious disease transmission.<br>Injury risk increases from aggressive behaviour. |
| | **4** | Inability to escape threats from unfamiliar conspecifics.<br>Increased potential for indirect aggressive behaviour from unfamiliar neighbours (food competition).<br>None of the general stress reduction due to presence of familiar conspecifics. |
| **4** | Group – with familiar companions – penned as a group.<br>**Assumptions:** Forage provided on floor prior to loading; not feral/wild (e.g., Shire horses, stock horses, rodeo horses).<br>**DOMAIN** | |
| | **1** | Restriction on water intake.<br>Competition for food resource leading to no access to food. |
| | **2** | Higher ammonia concentrations relative to individual transportation.<br>Increased space/ability to move around may be accompanied by increased ability to slip. |
| | **3** | Increased risk of injury from other horses; movement of vehicle may cause injuries.<br>Attempts to balance and adjust position may increase injury rate. |
| | **4** | Full social contact.<br>Competition for food resource.<br>General stress reduction due to presence of familiar conspecifics. |

**Table 11.** Assumptions and notes made when panellists considered interventions related to competition.

| **Duration** | Hours (assessment is of the equivalent of one day's training or competition). |
|---|---|

Pre-workshop assumptions: Assume horses are physiologically and behaviourally fit-for-purpose and level of activity; no riders >15% horse's bodyweight; rules (such as they are) are being observed and that aversive stimuli are being used according to the ISES Position Statement on Aversive Stimuli; assume horses are not wearing tongue ties or restrictive nosebands; duration of activity/day is adequate for fitness and regular competition for that activity.
Duration is in hours and the Frequency is at least once.

| | **Assumptions** |
|---|---|
| a | Horses are physiologically and behaviourally fit-for-purpose and for the level of activity. |
| b | Assessment is done for a trained horse on a single day of competition. |
| c | Rules (such as they are) are being observed. |
| d | All activities are assumed to be conducted at affiliated level. |
| e | Duration of activity/day is adequate for fitness and regular competition for that activity. |
| f | Competitors in each given sport are reasonably competent in application of negative reinforcement. |
| g | Assessment does not include housing/nutrition/transport conditions.<br>****Note:** Different sports have very different dietary regimes and housing. |

| | **Notes** | |
|---|---|---|
| **2** | Showing – in-hand.<br>**DOMAIN** | |
| | **1** | Horse may have limited control over feeding and drinking times. |
| | **2** | Novel and varied environment, new people, noises, public address (PA) systems, horses tied to trailers or lorries for periods of varying duration, transported, variable |

| | | |
|---|---|---|
| | | standards of safety of facilities at venues. Tack and equipment very variable, volunteer event managers with varying levels of knowledge. |
| | **3** | Metabolic disorders may arise due to chronic overfeeding, loss of thermal control and possible loss of proprioception due to rugging. |
| | **4** | Challenges include restraint, neophobia, thermo-regulatory challenges, confusion, unpredictable signals, horse conflicted, horses being chased, intimidation and excessive lungeing. |
| **3** | Showing – ridden.<br>**Note:** Not within rules but note documented use of inflammatory agents to induce elevated movements, use of weighted shoes to affect gait and irritants to affect tail height.<br>**DOMAIN** | |
| | **1** | Horse may have limited control over feeding and drinking times. |
| | **2** | As above, for showing in-hand. |
| | **3** | Aversive stimuli may be imposed via bits, spurs and whips. |
| | **4** | Unfamiliar riders (i.e., judges). |
| **4** | Eventing – competition.<br>**Assumptions:** As for dressage, plus kit for cross-country phase, including boots and martingales.<br>**DOMAIN** | |
| | **1** | Intermittent feeding and drinking opportunities. |
| | **2** | As above, for showing in-hand. |
| | **3** | Greater physical demand than during training, risk of over-heating, no control over start time to suit weather conditions such as ambient temperature.<br>As for dressage, but more varied activity. Risk of more severe injury is much higher than in dressage but the risk of repetitive strain injury is lower. |
| | **4** | Variety of training devices used during competition. Punishment can be common (e.g., when horse refuses). Continually exposed to novel stimuli, purposeful exposure to challenging situations (e.g., light/dark contrast, large jumps and ditches). |
| **6** | Dressage.<br>**Assumptions:** Appropriate footing substrate for competition, physical check by steward, veterinary inspection at highest levels, some control on use of medications; same rider, no restrictions on rider weight, likely to use spurs, dressage whip, double bridle (curb bit) optional from elementary level but mandatory at advanced level.<br>**DOMAIN** | |
| | **1** | Horse may have limited control over feeding and drinking times. |
| | **2** | As above, for showing in-hand. |
| | **3** | Repeated performance of similar movements could affect musculoskeletal health, performance of non-natural postures (hyper-flexion) could affect musculoskeletal and respiratory health. Exposure to dust from arena substrates. Potential for development of gait abnormalities including bridle lameness. High bit pressures may be applied relentlessly. Prevalence of gastric ulcers, may be high but is probably confounded with housing practices. |
| | **4** | Rider more aroused than during training thus increasing the risk of altered signalling and responses.<br>**Note:** Prevalence of stereotypies may be high but is probably confounded with housing practices. High degree of concentration required to respond to simultaneous/multiple signals – impact not yet known. Punishment can be common (e.g., when horse does not want to enter ring). |

| | | |
|---|---|---|
| **8** | Endurance.<br>**Assumptions:** Competition. No spurs, no whips >75cm, no whips in last phase of race, pre-ride vetting, 7 years-of-age minimum, most events will be 65-80km, but occasionally (3 times/year) 160km, horses are crewed, some rider variation (possible national variation in this).<br>**DOMAIN** | |
| | **1** | Horse may have limited control over feeding and drinking times. |
| | **2** | As above, for showing in-hand. Terrain may be very variable and often stony. |
| | **3** | Lameness (bruising most common), tendons, fatigue, metabolic disorders (colic), dehydration. |
| | **4** | Could be stabled in unfamiliar venue ahead of longer races. |
| **9** | Trail riding.<br>**Assumptions:** Hacking on own horse, up to 1 hour, no rules, unlikely to be supervised, very variable tack.<br>**DOMAIN** | |
| | **1** | – |
| | **2** | Very variable, unpredictable (e.g., with exposure to dogs and traffic), riders may be inexperienced. |
| | **3** | – |
| | **4** | – |
| **12** | Western performance (e.g., reining).<br>**Assumptions:** Competition – training can start at <2 years of age, competing at 3 years of age, rules according to FEI, severe bits and spurs but no whips, romal reins (leather rein with free loose end originally used in moving cattle but sometimes used to hit horse) may be used. Tests are short.<br>**DOMAIN** | |
| | **1** | – |
| | **2** | As above, for showing in-hand. Deep arena substrate. |
| | **3** | Musculoskeletal challenges resulting from high-speed acceleration, deceleration, turns, risk of injury from severe bits and sudden stops. Sharp rein pulls may be used and severe spurs with rowels. |
| | **4** | – |
| **13** | Community clubs (pony club, riding club).<br>**Assumptions:** Riders training and competing at unaffiliated level, show-jumping, cross-country and dressage. Horse must be 4 years old, rule books variable. No veterinary inspections or defined policies.<br>**DOMAIN** | |
| | **1** | – |
| | **2** | As above, for showing in-hand. Tack and equipment very variable, volunteer event managers. |
| | **3** | Musculoskeletal challenges due to inexperienced riders, infectious disease risk, biosecurity risks, transport effects on health, injury risks, fatigue. (? Perception among some that water deprivation might calm horse). |
| | **4** | Horse has limited control over feeding and drinking times, neophobia, thermo-regulatory challenges, confusion and unpredictable signals. Punishment can be common (e.g., when horse refuses). |
| **17** | Show jumping. | |

| | | |
|---|---|---|
| | **Assumptions:** National federation rules (derived from the FEI) for major events – here we assume compliance with national federation rules, horses are trained to jump type of obstacles likely to encounter.<br>**DOMAIN** | |
| | **1** | – |
| | **2** | As above, for showing in-hand. Variable footing. Tack and equipment very variable, spurs, restrictive nosebands, martingales and severe bits very common, whips common (some rules about whip length). |
| | **3** | Musculoskeletal challenges, infectious disease risk, biosecurity risks, transport effects on health, injury risks, fatigue. Elevated injury risk due to concussive forces on joints and limbs. |
| | **4** | Restraint. Horse has limited control over feeding and drinking times, neophobia, thermo-regulatory challenges, confusion and unpredictable signals. Punishment can be common (e.g., when horse refuses).<br>Extreme lateral neck flexion as warm-up. |
| **20** | Flat racing.<br>**Assumptions:** Competition – horse familiar with race environment, including start gates, national federation rules apply, horses race from 2 years of age, 1000–3600m distance, padded whips carried and used, small spurs. Some veterinary inspection of horses on ground (stalls, swab box, track), no routine individual trot-ups.<br>**DOMAIN** | |
| | **1** | – |
| | **2** | Variable footing from slippery or impacted surfaces, variable crowd conditions, possibility of use of other equipment to stimulate horse to race and to focus their efforts (e.g., blinkers). |
| | **3** | Elevated injury risk due to concussive force on limbs and joints, especially for younger horses.<br>Racing plates being frequently replaced can weaken hoof wall.<br>Likely to train and race in one direction with some asymmetry possible.<br>Some thermo-regulatory challenges. |
| | **4** | Riders may use the whip for positive punishment.<br>There may be variable rein tension.<br>There may be some panic reactions.<br>Tongue ties commonly restrict tongue movement. |
| **22** | Jumps racing.<br>**Assumptions:** Competition – as for flat racing but horses are older (4 years). Horses have been trained to jump obstacles.<br>**DOMAIN** | |
| | **1** | – |
| | **2** | Variable footing from slippery or impacted surfaces, variable crowd conditions, possibility of use of other equipment to stimulate horse to race and to focus their efforts (e.g., blinkers). |
| | **3** | As above for flat racing but with elevated injury risk to legs, risk of falls and fatalities. |
| | **4** | Interference from other horses can lead to collisions or falls. Tongue ties often used, restricting tongue movement. |
| **24** | Harness racing.<br>**Assumptions:** Competition – horses familiar with racing environment including starting barriers, rules abided by, horses have been trained to pull sulkies, can start racing at 2 years of age but training can begin before the horse is 2 years. | |

| | | |
|---|---|---|
| | **DOMAIN** | |
| | **1** | – |
| | **2** | Footing may be firm/hard. |
| | **3** | Risk of injury due to concussive forces on limbs and joints, risk of accidents, (including collisions), travel in one direction. |
| | **4** | Punishment with whips, head restraints/checks, burrs, blinkers, hobbles for pacers and lugging poles. Tongue ties commonly used, restricting tongue movement. |
| **26** | Polo.<br>**Assumptions:** Competition – abides by regulations of the sport, no sharp spurs, removal from game if blood observed, after fall of pony trotted-up before remounting, lame horses removed, minimum age 4 years.<br>**DOMAIN** | |
| | **1** | – |
| | **2** | Variable footing. Some variability in environment, unfamiliar riders and rider weight. |
| | **3** | Elevated injury risk due to high-speed acceleration, deceleration, turns and impacts. Risk of physical trauma between horses, risk of injury from mallets, 7-minute chukkas mean that physical exertion is of very high intensity. |
| | **4** | Use of restrictive equipment is common. |

**Table S12.** Assumptions and notes made when panellists considered interventions related to work.

| | |
|---|---|
| **Duration** | Hours (assessment is based on the equivalent of one day's work). |

| | **Assumptions** |
|---|---|
| a | Assessment does not include housing/nutrition/transport conditions. |
| b | Duration of activity per day is adequate for fitness and regular work. |
| c | Arena surface is safe, suitable, and well managed. |
| d | Tack is clean and well-fitting. |
| e | Rider is well matched to the horse's size and weight. |

| | **Notes** | |
|---|---|---|
| **1** | Riding school/Riding for the Disabled/Therapeutic riding horses.<br>**Assumptions:** Each horse is being coached, horse and rider are well matched.<br>**DOMAIN** | |
| | **1** | – |
| | **2** | Barren work environment (arena). |
| | **3** | Potential for chronic low-level lameness (especially among older horses), intermittent exercise and potential for reliance on the use of whips. |
| | **4** | Unpredictable, intermittent and/or conflicting pressures via legs and reins. General unpredictability in environment.<br>Changing composition of horse groupings during lessons. |
| **2** | Rental stables (unsupervised). | |

| | | |
|---|---|---|
| | **DOMAIN** | |
| | **1** | – |
| | **2** | Varying environments and climatic conditions.<br>**Note:** Best practice may be difficult to maintain due to varying conditions/riding – no supervision to detect issues. |
| | **3** | Lameness, injuries, saddle sores, gastric ulcers, over-riding (reduced monitoring and interventions). |
| | **4** | Varying standards of riding and varying duration of ridden sessions.<br>Unpredictable events.<br>Changing composition of horse groupings within and between lessons. Unpredictable, intermittent and/or conflicting pressures via legs and reins. |
| **4** | Stock work.<br>**DOMAIN** | |
| | **1** | **Note:** Restricted access to water and graze during work. |
| | **2** | Varied environment, working for different lengths of time over rough, unpredictable terrain.<br>The use of quad bikes and horses may be combined. |
| | **3** | Seasonal work may be associated with lack of fitness, tying-up, injuries, sores and exhaustion. |
| | **4** | Grouping of horses may change. |
| **5** | Police work.<br>**DOMAIN** | |
| | **1** | – |
| | **2** | Environment unpredictable and may include challenges such as noise and smoke.<br>Working surface may be not ideal for prolonged work. |
| | **3** | Dangerous environment brings with it an elevated risk of injury. |
| | **4** | May encounter novel stimuli that they find aversive.<br>May not have fully habituated to all stimuli they encounter regularly. |
| **6** | Carriage and haulage.<br>**DOMAIN** | |
| | **1** | Restricted access to food and water. |
| | **2** | Variation in the duration of work.<br>Restricted choice of access to shelter or shade.<br>Working surface may not be ideal for prolonged work.<br>Wearing a lot of tack amounts to extra weight being carried.<br>Pollutants may be inhaled. |
| | **3** | Lameness may arise from accumulation of concussive interactions with metalled roads.<br>Unpredictable environment, especially with other vehicular traffic, may elevate the risk of injury. |
| | **4** | Inability to escape aversive stimuli. |
| **7** | Rodeo horses.<br>**Assumption:** Process aversive but some level of controllability and predictability during work.<br>**Note:** Rodeo Code of Practice available at (http://nationalrodeoassociation.com.au/13site/rulebook2006.pdf) but we noted that this is not followed at all rodeos.<br>**DOMAIN** | |

| | | |
|---|---|---|
| | **1** | Potential limited water-trough access in yards prior to work. |
| | **2** | Constantly changing and novel environments.<br>When not working they are being transported or may be confined.<br>Reduced frequency of work compared with other working activities. |
| | **3** | Risk of musculoskeletal injuries. |
| | **4** | When not working, housed with other horses that may include some social flux.<br>Duration of work very short (less than 8 seconds).<br>Possible fear response in chute.<br>Unpredictable working environment with unfamiliar people around chute and the auditory challenges from the public address (PA) system.<br>Aversive stimuli may include spurring on shoulders. |
| **8** | Pregnant Mare's Urine (PMU) ranching (urine collection).<br>**Note:** Working season – housed in open-tie stalls in barns for 6 months. Turned-out for short periods every 2 weeks.<br>During off season, mares occupy open pasture for 6 months.<br>**Assumptions:** Assessed only during 6 months of indoor work.<br>Constant access to water and roughage.<br>Concentrate rations supply appropriate vitamins and minerals.<br>Equipment for urine collection is in contact with the mares only when they urinate.<br>Good barn hygiene minimises ammonia concentrations in the inhaled air.<br>**DOMAIN** | |
| | **1** | – |
| | **2** | Some noise in barn due to biosecurity equipment.<br>Barren environment. |
| | **3** | – |
| | **4** | Able to lie down and get up but cannot turn around.<br>Social contact compromised but horses standing to either side can be physically contacted. |

**Table S13.** Assumptions and notes made when the panellists considered interventions related to breeding mares.

| | |
|---|---|
| **Duration** | Days to weeks – duration of one breeding cycle – to pregnancy. |

Pre-workshop assumptions: Duration is in weeks and the frequency is at least once.

| | **Assumptions** |
|---|---|
| a | Includes all interventions that accompany a potential breeding cycle. |
| b | Healthy and breeding-sound mares. |
| c | Mating occurs when the mare is in oestrus. |
| d | Assume no foal at foot. |
| e | Breeding area: Good footing, adequate ceiling height, adequate lighting, clean. |
| f | Procedures are undertaken by skilled technicians. |
| g | For **artificial insemination** and **in-hand matings:**<br>On stud farm – no artificial lighting.<br>Exogenous hormones administered for stimulation of oestrus and/or ovulation. |

| | **Notes** |
|---|---|

<table>
<tr><td rowspan="5">1</td><td colspan="2">Broodmare – pasture matings.<br>Assumptions: Pasture has safe (fencing, footing, etc.) and the enclosure is of adequate size for the group.<br>Band of mares familiar with each other prior to introducing to stallion.<br>Stallion experienced with pasture breeding.<br>DOMAIN</td></tr>
<tr><td>1</td><td>–</td></tr>
<tr><td>2</td><td>–</td></tr>
<tr><td>3</td><td>Venereal disease, breeding accidents, vaginal injuries, kick, bite, perineal injuries, (low risk), infectious disease.</td></tr>
<tr><td>4</td><td>Introduction of stallion might cause disruption, increased locomotion, no choice of mate.</td></tr>
<tr><td rowspan="5">2</td><td colspan="2">Broodmare – in-hand matings.<br>Assumptions: Oestrus (peri-ovulatory) is confirmed with rectal ultrasound exam and behaviour observations (teased and in standing heat).<br>Restraint – Best practice: Safe stocks (crush) for exam and safe environment for teasing. At the time of mating: Hobbles, breeding cape, tail wrap, nose twitch, halter with stud chain over the nose.<br>Mare and stallion genitals are washed.<br>DOMAIN</td></tr>
<tr><td>1</td><td>–</td></tr>
<tr><td>2</td><td>Moved from various areas to breeding environment, artificial environment.</td></tr>
<tr><td>3</td><td>Increased risk of disruption to commensal flora due to washing, decreased risk of venereal disease, increased risk of injury due to slipping, falling, restraint, increased risk of injury from stallion.</td></tr>
<tr><td>4</td><td>Residual olfactory stimuli, oestrus overridden by pain or stress from restraint. No pre-, peri- and post-copulatory behaviours nor any choice about breeding time, event, mate or location. Possibility of punishment for repulsion behaviours.</td></tr>
<tr><td rowspan="5">3</td><td colspan="2">Broodmare – artificial insemination.<br>Assumptions: Teasing and cycle management as for in-hand matings.<br>Fresh or cooled transported (less management than frozen) semen.<br>Restraint: Minimal (stocks or stall).<br>DOMAIN</td></tr>
<tr><td>1</td><td>–</td></tr>
<tr><td>2</td><td>–</td></tr>
<tr><td>3</td><td>Low risk of injury and infection, very minimal risk of venereal disease.</td></tr>
<tr><td>4</td><td>No pre-, peri- and post-copulatory behaviours nor any choice about breeding time, event, mate or location.</td></tr>
<tr><td rowspan="3">6</td><td colspan="2">Wet nurse (transition from one foal to another).*<br>Assumptions: Draught mare, young, good-quality/quality milk is given to the foal, industry of providing wet nurses.<br>Within 72 hours post-partum, restrained in a box stall during removal from her foal, transported to the stud farm and new foal is introduced through a nursing gate.<br>Transport less than one hour.<br>Assumptions: Acceptance of non-biological foal within a few hours.<br>DOMAIN</td></tr>
<tr><td>1</td><td>Some impact of feeding and drinking.</td></tr>
<tr><td>2</td><td>Unfamiliar environment, confinement.</td></tr>
</table>

| | | |
|---|---|---|
| | **3** | Infectious disease risk high, risk of injury and risk of mastitis. |
| | **4** | Removal from social partners and foal*, potential foal rejection, punishment for foal rejection, sedation, restraint,<br>*impact of weaning on foals is considered elsewhere. |

**Table S14.** Assumptions and notes made when panellists considered interventions related to breeding stallions.

| | |
|---|---|
| **Duration** | Hours (assess impact of activity in one day). |

Pre-workshop assumptions: Duration is in hours and the frequency is at least once.

| | **Assumptions** |
|---|---|
| a | Assume mare or dummy mare is safe and not over-used. |
| b | Stallion is reasonably amenable to handling. |
| c | Stallion has had positive previous exposure to mares in the given situation (i.e., appropriate experience to court and mate in that environment). |
| d | Stallion is fit, healthy and disease-free. |
| e | Competent handlers (acknowledging that normal practice is not best practice). |
| f | Underfoot substrate reduces risk of slipping. |
| | **Note:** Have not assessed general issues associated with stallion housing and management. |

| | **Notes** | |
|---|---|---|
| **1A** | Breeding stallion – in-hand matings.<br>**Assumptions:** Low noise, high ceiling, appropriate lighting, safe environment; reasonable measures to prevent injury.<br>**Note:** This assessment could also apply to semen collection with a dummy mare. | |
| | **1** | _ |
| | **2** | _ |
| | **3** | Risk of venereal disease.<br>High risk of injury from mare and as a result of excitable behaviour (e.g., hyperextension of a limb, penile trauma, getting caught up in mare's hobble).<br>Very low risk of scrotal hernia.<br>Very low risk of aortic rupture or other cardiac event. |
| | **4** | Mating experience may increase difficulties in handling around mares.<br>Removal of access to mares may result in frustration.<br>Thwarted mating events may result in frustration.<br>Inability to perform pre- and post-copulatory behaviour.<br>Inability to fully assess mare's receptivity.<br>Possible challenges from residual olfactory stimuli (e.g., if multiple stallions serve mares in the same area).<br>Risk of being punished to reduce behaviours (e.g., vocalisation, rushing ahead, striking, rearing, staying with mare. |
| **1B** | Breeding stallion – AI semen collection with a dummy mare.<br>**Assumptions:** Low noise, high ceiling, appropriate lighting, safe enclosure; well-managed stimulus mare present.<br>**DOMAIN** | |
| | **1** | – |

| | | |
|---|---|---|
| | **2** | – |
| | **3** | Very low to zero risk of venereal disease.<br>Risk of injury from dismount behaviour (e.g., hyperextension of a limb).<br>Increased risk of penile trauma from dummy mare.<br>Very low risk of scrotal hernia.<br>Very low risk of aortic rupture or other cardiac event. |
| | **4** | Mating experience may increase difficulties in handling around mares.<br>Lack of access to stimulus mare may result in frustration.<br>Inability to perform pre- and post-copulatory behaviour.<br>Possible challenges from residual olfactory stimuli.<br>Risk of being punished to reduce behaviours (e.g., vocalisation, rushing ahead, striking, rearing). |
| **3** | Breeding stallion – pasture matings.<br>**Assumptions:** Stallion is kept for extended time with mares.<br>**DOMAIN** | |
| | **1** | Reduced voluntary feed intake. |
| | **2** | – |
| | **3** | Risk of venereal disease.<br>Some injury risk from mares (stallion has capacity to exercise choice).<br>Very low risk of scrotal hernia.<br>Very low risk of aortic rupture or other cardiac event.<br>Lost condition due to reduced feed intake. |
| | **4** | Access to multiple mares.<br>Mares are familiar – social relationships already established.<br>Ability to perform pre- and post-copulatory behaviour.<br>Ability to fully assess mare’s receptivity. |
| **4** | Teaser horse.<br>**Assumptions:** Good management includes occasional copulation; includes stallions that perform oestrus detection and those that just perform test mounts; reasonable measures to prevent injury.<br>**DOMAIN** | |
| | **1** | – |
| | **2** | – |
| | **3** | Higher risk of injury from mare as a result of excitable behaviour (e.g., hyperextension of a limb, penile trauma, getting caught up in mare’s hobble).<br>Risk of penile trauma. |
| | **4** | Mating experience may increase difficulties in handling around mares.<br>Lack of access to stimulus mare may result in frustration.<br>Inability to perform pre- and post-copulatory behaviour.<br>Higher risk of being punished to reduce behaviours (e.g., vocalisation, rushing ahead, striking and rearing) (teaser horses are generally less well handled than breeding stallions). |
